# *Staphylococcus aureus* toxin LukSF dissociates from its membrane receptor target to enable renewed ligand sequestration

**DOI:** 10.1096/fj.201801910R

**Published:** 2018-12-03

**Authors:** Karita Haapasalo, Adam J. M. Wollman, Carla J. C. de Haas, Kok P. M. van Kessel, Jos A. G. van Strijp, Mark C. Leake

**Affiliations:** * Department of Medical Microbiology, University Medical Center Utrecht, Utrecht University, Utrecht, The Netherlands;; † Department of Bacteriology and Immunology, University of Helsinki, Helsinki, Finland;; ‡ Department of Biology, Biological Physical Sciences Institute, University of York, York, United Kingdom;; ‖ Department of Physics, Biological Physical Sciences Institute, University of York, York, United Kingdom

**Keywords:** bacterial toxin, pore formation, single molecule, super-resolution, immune response

## Abstract

*Staphylococcus aureus* Panton-Valentine leukocidin is a pore-forming toxin targeting the human C5a receptor (hC5aR), enabling this pathogen to battle the immune response by destroying phagocytes through targeted lysis. The mechanisms that contribute to rapid cell lysis are largely unexplored. Here, we show that cell lysis may be enabled by a process of toxins targeting receptor clusters and present indirect evidence for receptor “recycling” that allows multiple toxin pores to be formed close together. With the use of live cell single-molecule super-resolution imaging, Förster resonance energy transfer and nanoscale total internal reflection fluorescence colocalization microscopy, we visualized toxin pore formation in the presence of its natural docking ligand. We demonstrate disassociation of hC5aR from toxin complexes and simultaneous binding of new ligands. This effect may free mobile receptors to amplify hyperinflammatory reactions in early stages of microbial infections and have implications for several other similar bicomponent toxins and the design of new antibiotics.—Haapasalo, K., Wollman, A. J. M., de Haas, C. J. C., van Kessel, K. P. M., van Strijp, J. A. G., Leake, M. C. *Staphylococcus aureus* toxin LukSF dissociates from its membrane receptor target to enable renewed ligand sequestration.

*Staphylococcus aureus* causes diseases ranging from superficial skin and soft tissue infections to severe invasive diseases, such as osteomyelitis and necrotizing pneumonia (1). During the 1960s, methicillin-resistant *S. aureus* (MRSA) was identified as a nosocomial pathogen (2). In the 1990s, infection of previously healthy, community-dwelling individuals with MRSA was reported (3). Since then, these community-associated MRSA have rapidly emerged worldwide (4). Variants have also recently been identified that have reduced susceptibility to the antibiotic vancomycin (5), as well as complete resistance (6), and these forms of *S. aureus* pose a significant threat to human health. S. *aureus* and resistant variants have also evolved adaptations to evade attack from cells of the human immune system. However, the molecular processes that underlie these strategies are underexplored in living cells. There are compelling scientific and societal motivations to understand the mechanisms involved in immunogenic evasion strategies of *S. aureus*.

In the early 1930s, Panton and Valentine described a powerful leukocidal toxin produced by multiple *S. aureus* isolates, now denoted Panton-Valentine leukocidin (Luk; PVL), years later shown to be cytotoxic to neutrophils, monocytes, and macrophages but not to lymphocytes (7, 8). The majority of community-associated MRSA isolates carry the genes encoding PVL, partially as a result of the successful spread of the PVL carrying clone USA300 in the United States (3, 4, 9, 10), rarely present in hospital-acquired antimicrobial-resistant MRSA and methicillin-susceptible *S. aureus* isolates. Based on epidemiologic studies, PVL is associated with primary skin infections in humans, osteomyelitis, and in particular, severe necrotizing pneumonia (11, 12). Necrotizing pneumonia is a severe complication caused by bacterial lung infection. It is characterized by massive recruitment of neutrophils in the site of infection, diffuse pulmonary inflammation, septic shock, and respiratory failure. Both host factors and microbial virulence factors are thought to play an important role in the inflammation; however, it is unknown how the interplay between these 2 factors affects the severity of the disease (13). The specificity to cell-surface receptors makes it difficult to study the role of PVL in *S. aureus* pathogenesis in a whole animal model. It is possible that lysis of neutrophils by PVL is responsible for a reduced host defense response allowing the pathogen to spread and cause eventual tissue damage. However, a previous study using a rabbit animal model on necrotizing pneumonia suggests that PVL itself directly or indirectly causes tissue injury and by this way, induces local inflammation (14).

PVL is a prophage-encoded bicomponent, β-barrel pore-forming toxin (PFT) comprising protein subunits Luk components S and F (LukS and LukF, respectively). Binding of LukS and LukF to the surface of target cells induces formation of the pore; chemical and genetic analysis suggests that the resulting complex consists of a lytic pore-forming hetero-octamer (15, 16). Stoichiometric analysis *in vitro* of this complex suggests it is an octamer of 4-plus-4 subunits (17). In this complex, only LukS is known to interact with the human C5a receptor [hC5aR; cluster of differentiation 88 (CD88)], a 7-transmembrane GPCR. LukS targets at least the extracellular N terminus of hC5aR (18, 19), similar to the chemotaxis inhibitory protein of *S. aureus*, but may also interact with the transmembrane receptor region (20). C5a is a powerful anaphylatoxin released during complement activation that is a powerful first-line defense mechanism against invading pathogens. Activation of complement on the target leads to a rapid opsonization with C3b (21). Further activation of complement leads to formation of C5a and membrane attack complexes that are lytic for Gram-negative but not Gram-positive bacteria (22, 23). Therefore, in defense against Gram-positive bacteria, C3b opsonization, together with attraction and activation of neutrophils *via* C5a–C5aR interaction, is essential (24, 25). In severe cases, formation of C5a can potentially lead to hyperactivation of the inflammatory response, an inability to regulate this potentially fatal reaction, and eventually harm the human host tissues. Because of this strong proinflammatory activity, therapeutic interventions have recently focused on neutralizing antibodies against C5a and C5aR as potential candidates for the treatment of severe inflammatory conditions, such as bacterial-induced sepsis (26, 27).

LukS binding to hC5aR inhibits C5aR binding, which efficiently blocks neutrophil activation (18). LukS receptor binding alone is not sufficient for cell lysis but requires simultaneous interaction between the Luk subunits and hC5aR. However, multiple possible subunit and receptor combinations are theoretically possible, and the spatiotemporal dynamics in functional complexes in live cells among LukS, LukF, and hC5aR is not yet known. In addition to PVL, *S. aureus* can produce a number of other β-barrel PFTs with varying receptor and cell-type specificities from which most of them are classified as bicomponent toxins, such as PVL (28).

Development of methods to study dynamic processes of pore formation by these toxins at a molecular level may improve our understanding of the evolution of bacterial virulence and human immunity. There are several studies that have attempted to explain the function of bacterial PFTs, including structural and subunit stoichiometry data from high-resolution X-ray crystallography and single-molecule fluorescence microscopy (17, 29, 30). However, these studies focused on pathogen instead of host factors and were thereby limited in excluding the specific interaction between host cell receptor and bacterial toxin component, the first step required for toxin oligomerization on the host cell membrane and the presence of the most potent factor mediating the inflammatory response *via* C5a recognition in the site of infection (18).

Here, we used standard and single-molecule fluorescence detection with super-resolution localization microscopy (31) to determine protein complex assembly on receptors in live and fixed cell membranes. We studied human embryonic kidney (HEK) cells modified to express monomeric green fluorescent protein (mGFP)-labeled hC5aR, exposed to Alexa Fluor dye-labeled *S. aureus* toxin components LukS and LukF and imaged using standard total internal reflection fluorescence (TIRF) real-time microscopy (Supplemental Fig. S1), allowing us to monitor the spatiotemporal dynamics of receptor and toxin molecules in the cell membrane. Our findings indicate that LukS binds on clusters of membrane-integrated hC5aRs. The receptor-bound LukS then binds LukF leading to the formation of a pore that is consistent with previous stoichiometric studies. However, when LukF is bound to the complex, we observe fewer colocalized hC5aRs with toxin in fixed cells, more immobilized toxin complexes in live cells, and a significantly reduced Förster resonance energy transfer (FRET) signal, indicating, unexpectedly, that pore formation leads to simultaneous dissociation of the receptors from the complex. In addition, our biochemical data suggest that the dissociated receptor can then be available for additional LukS molecules or the C5a generated during complement activation as a response to LukS and LukF (LukSF)-mediated cell lysis. Although indirect evidence, this new finding suggests that a limited number of receptors can be “recycled” as docking for further toxin. This ensures that a sufficient number of pores will damage nearby phagocytic cells, particularly important when high numbers of C5a anaphylatoxin are blocking LukS, and potentially also enables a simultaneous C5a-mediated inflammatory response on adjacent cells.

## MATERIALS AND METHODS

### Experimental model and subject details

#### PMN isolation, cell lines, and transfections

Human blood was obtained from healthy volunteers, and the polymorphonuclear (PMN) cells were isolated by Ficoll/Histopaque centrifugation (32). Informed consent was obtained from all subjects in accordance with the Declaration of Helsinki and the Medical Ethics Committee of the University Medical Center Utrecht (METC-protocol 07-125/C; approved March 1, 2010). To ensure a truly monomeric state and prevent GFP-mediated clustering of the receptor, a fusion construct of hC5aR with the mGFP variant with the A206K mutation (also denoted GFPmut3) (33, 34) was made at the C terminus (primers used listed in **Table 1**; source data 2), or a sortase A lysine-proline-x-threonine-glycine-glycine (LPXTGG) sequence was made in the N terminus and cloned into plasmid IRES puromycin (pIRESpuro) vectors (Table 1) by PCR. The amplification reaction was performed in 3 separate amplification steps using overlap extension PCR on hC5aR and mGFP templates. hC5aR (accession number of hC5aR = NM_00173) was used as the template using enzymes and purification kits, as previously described. The clones were ligated into the vectors and transferred into TOP10 *Escherichia coli*-competent cells and then amplified and sequenced similarly to the toxin clones previously described. The pIRESpuro/hC5aR-mGFP vector was transfected into HEK 293T cells (a HEK cell line; Thermo Fisher Scientific, Waltham, MA, USA), stably expressing G protein Gα16, using Lipofectamine-2000 reagent, according to the manufacturer’s instructions (Thermo Fisher Scientific). After 24–48 h, transfected cells were harvested with 0.05% trypsin. To obtain a uniform, stable culture, cells were subcloned in a concentration of 0.5 cell/well in a 96-well plate in DMEM (Lonza, Basel, Switzerland), supplemented with 10% fetal calf serum (Thermo Fisher Scientific) 100 U/ml penicillin/100 µg/ml streptomycin (Thermo Fisher Scientific), 1 µg/ml hygromycin, and 250 µg/ml puromycin. For N-terminal labeling of the sortase A recognition sequence containing HEK cells with FITC were successfully performed in 2 steps as previously described (35). The THP-1 human monocytic cell line was grown in Roswell Park Memorial Institute (RPMI) 1640 medium (Thermo Fisher Scientific), supplemented with 1 time Glutamax (Thermo Fisher Scientific), 10% fetal calf serum (Thermo Fisher Scientific), 100 U/ml penicillin, 100 µg/ml streptomycin (Thermo Fisher Scientific), and 25 mM HEPES (Thermo Fisher Scientific). Differentiation of THP-1 monocytes into macrophages was done by the incubation of cells in the medium for 48 h with 100 ng/ml phorbol 12-myristate 13-acetate. Expression of hC5aR was analyzed by the incubation of cells in 50 µl RPMI (Thermo Fisher Scientific), supplemented with 0.05% human serum albumin (HSA; Sanquin, Amsterdam, The Netherlands), RPMI-HSA at 5 × 10^6^ cell/ml concentration for 45 min with phycoerythrin (PE)-conjugated anti-CD88 (BD Biosciences, San Jose, CA, USA), and detected by flow cytometry. The presence of mGFP or FITC-LPXTG was detected directly by flow cytometry.

**TABLE 1:** Cloning sequence details

Clone (restriction site)	gBlock primer, 5′–3′
3′mGFP(*Not*I)stop	ATATGCGGCCGCTTATTTGTATAGTTCATCCATG
5′KOZ-hC5aR(*Bam*HI)	ATATGGATCCGCCGCCACCATGAACTCCTTCAATTATAC
5′hC5aR-mGFP	AGACCCAGGCAGTGAGTAAAGGAGAAGAACTTTTC
3′hC5aR-mGFP	TCTCCTTTACTCACTGCCTGGGTCTTCTGGGCCATAG
5′N-sor-hC5aR	CGGGATCCGCCGCCACCATGCTACCCGAGACTGGAGGCGGAGGTGGCAACTCCTTCAATTATACCAC
3′hC5aR-not	ATATGCGGCCGCCTACACTGCCTGGGTCTTCTG
5′LukF-K288C(*Bam*HI)	CGGGATCCGCTCAACATATCACACCTGTAAG
3′LukF-K288C(*Not*I)	ATATGCGGCCGCTTAGCTCATAGGATTTTTTTCCTTAGATTGAGTATCTATTAAGCAAACTGTATGATTTTCCCAATC
3′LukS-K281C(*Not*I)	ATATGCGGCCGCTCAATTATGTCCTTTCACGCAAATTTCATGAGTTTTCC
5′LukS-Y113H	GTCAAACATTAGGTCATAACATAGGTGGTAATTTTAATAG
3′LukS-Y113H	TTACCACCTATGTTATGACCTAATGTTTGACTAAC
5′LukS(*Bam*HI)	CGGGATCCAAAGCTGATAACAATATTGAG
F(G130D) gBlock *Bam*HI/*Not*I pRSET B C-his overlap	CTTTAAGAAGGAGATATACATATGGGATCCCAACATATCACACCTGTAAGTGAGAAAAAGGTTGATGATAAAATTACTTTGTACAAAACAACTGCAACATCAGATTCCGATAAGTTAAAAATTTCTCAGATTTTAACTTTTAATTTTATTAAAGATAAAAGTTATGATAAAGATACATTAATACTCAAAGCTGCTGGAAACATTTATTCTGGCTATACAAAGCCAAATCCAAAAGACACTATTAGTTCTCAATTTTATTGGGGTTCTAAGTACAACATTTCAATTAATTCAGATTCTAATGACTCAGTAAACGTTGTAGATTATGCACCTAAAAATCAAAATGAAGAATTTCAAGTACAACAAACGGTAGGTTATTCTTATGGTGGAGATATTAATATCTCTAACGGCTTGTCAGGTGATGGTAATGGTTCAAAATCTTTTTCAGAGACAATTAACTATAAACAAGAAAGCTATAGAACTAGCTTAGATAAAAGAACTAATTTCAAAAAAATTGGTTGGGATGTTGAAGCACATAAAATTATGAATAATGGTTGGGGACCATATGGCAGAGATAGTTATCATTCAACTTATGGTAATGAAATGTTTTTAGGCTCAAGACAAAGCAACTTAAATGCTGGACAAAACTTCTTGGAATATCACAAAATGCCAGTGTTATCCAGAGGTAACTTCAATCCAGAATTTATTGGTGTCCTATCTCGAAAACAAAACGCTGCAAAAAAATCAAAAATTACTGTTACTTATCAAAGAGAAATGGATAGATATACAAACTTTTGGAATCAACTTCACTGGATAGGTAATAATTATAAAGATGAAAATAGAGCAACTCATACATCAATTTATGAAGTTGATTGGGAAAATCATACAGTTAAATTAATAGATACTCAATCTAAGGAAAAAAATCCTATGAGCGCGGCCGCACACCATCACCATCACCATTAA

#### Recombinant protein production and purification

Polyhistidine-tagged LukS and LukF were cloned and expressed using an *E. coli* expression system. For maleimide-based labeling, a single-cysteine mutation was designed to the LukS and LukF components [*S. aureus* Luk S mutant (mS) and *S. aureus* Luk F mutant (mF)] based on previous data and the crystal structure of the octameric pore (29). An additional mutation Y113H was included in LukS to facilitate oligomerization of the maleimide-labeled protein (17). The target genes were amplified by PCR (Table 1) from the wild-type sequences using Phusion High-Fidelity DNA polymerase (Thermo Fisher Scientific) (18). The PCR product was cloned into a slightly modified plasmid Restriction Enzyme T7 promoter (pRSET) expression vector (Thermo Fisher Scientific), resulting in expression of proteins with an N-terminal 6×HIS-tag. For LukF mutant G130D LukF [F(G130D)], we used a gBlock (a custom double-stranded DNA sequence *via* Integrated DNA Technologies, Coralville, IA, USA) to incorporate the LukF in the pRSET vector. Clones were sequenced to verify the correct sequence. The recombinant proteins were expressed in Rosetta-gami 2 (DE3) pLysS *E. coli* using 1 mM isopropylthio-β-galactoside induction and isolated by a native isolation method. The expressed proteins were purified according to the manufacturer’s instructions (Thermo Fisher Scientific) using 1 ml nickel HisTrap and Superdex 75 HiLoad columns (GE Healthcare Life Sciences, Marlborough, MA, USA). Toxin components were labeled with either Cy3 (GE Healthcare, Waukesha, WI, USA), Alexa Fluor 594 or Alexa Fluor 647 C_2_ maleimide reagent, according to the manufacturer’s instructions (Thermo Fisher Scientific), resulting in negligible unlabeled content. The labeling efficiency was 100%, as determined by protein concentrations using absorption at 280 nm and dye concentrations using absorption at 650 nm by a Nanodrop ND-1000 spectrophotometer.

### Method details

#### Binding assays

Binding of the maleimide-labeled proteins to PMN and HEK cells was confirmed by flow cytometry. LukS-K281C-Y113H (mS) or wild-type mS [S(wt)] was labeled with FITC, FITC-S(wt), or Alexa Fluor maleimide 647- or 594-labeled mS (mS*). For competition assays, 3 µg/ml of the labeled protein and increasing concentration of non mS* or S(wt) were incubated with isolated PMNs or HEK hC5aR-mGFP cells (5 × 10^6^ cell/ml) in a total volume of 50 µl RPMI-HSA on ice. For binding assays without competition, the cells were incubated with an increasing concentration of labeled mF (mF*). After 30 min incubation on ice, cells were washed, fixed with 1% paraformaldehyde, and analyzed by flow cytometry. HEK cells transfected with the C–C chemokine receptor type 2 (CCR2) receptor were used as negative control for mS binding. To see inhibition of PE-anti-CD88 (BD Biosciences) binding by S(wt) or C5a, hC5aR-expressing HEK cells were first incubated with increasing concentrations of S(wt) or C5a for 45 min at 4°C. Then, 2 µl anti-CD88/200,000 cells was added and incubated as previously described. Cells were washed once with RPMI-HSA, fixed with 1% paraformaldehyde, and analyzed by flow cytometry. To detect hC5aR dissociation using sublytic concentrations of LukSF, hC5aR-expressing HEK cells were incubated with 100 nM S(wt) for 45 min at 4°C. After washing the unbound S(wt) sublytic concentrations of wild type mF [F(wt)] were added to the cells and incubated for 20 min at 37°C and 5% CO_2_ atmosphere. Percentages of lysed *vs.* nonlysed cells were measured by using 1 µg/ml DAPI (MilliporeSigma, Burlington, MA, USA) in the reaction. For C5a-rebinding assay, 1 µM C5a (MilliporeSigma) was labeled with NT647, according to the manufacturer’s instructions (NanoTemper Technologies, Munich, Germany). Free label from the sample was removed by 3 times centrifugation through Amicon Ultra 0.5 ml centrifugal filters (MilliporeSigma). The hC5aR-expressing HEK cells were incubated with 1 µM S(wt) for 45 min at 4°C. After washing, 20 nM NT647-labeled C5a and increasing concentrations of F(wt) were added to the cells and incubated for 20 min at 37°C and 5% CO_2_ atmosphere. Cells were washed once with RPMI-HSA, fixed with 1% paraformaldehyde, and analyzed by flow cytometry. Percentages of lysed *vs.* nonlysed cells were measured by using 1 µg/ml DAPI in the reaction. *S. aureus* extracellular complement-binding (Ecb) protein was used as a negative control, as it interacts with another cell-surface receptor, CR1 (36). Flow cytometry data were analyzed using the FlowJo (Tree Star, Ashland, OR, USA) v.10 software package.

#### Cell-permeability assays

Isolated PMNs or HEK hC5aR-mGFP cells (5 × 10^6^ cell/ml) were exposed to labeled and unlabeled mixtures as appropriate for mF/mS recombinant proteins at equimolar concentrations in a volume of 50 µl RPMI-HSA with 1 µg/ml DAPI. Cells were incubated for 30 min at 37°C with 5% CO_2_ and subsequently analyzed by flow cytometry. To calculate the lysis time, cells were first incubated with 150 nM mS for 15 min. Then, 600 nM mF was added and immediately subjected to flow cytometry analysis, where the permeability was measured at several time points. Cell lysis was defined as intracellular staining by DAPI. HEK cells transfected with the human CCR2 receptor was used as a negative control for toxin-mediated lysis. Statistical differences between means of repeated experiments were calculated using 2-tailed Student *t* tests.

#### Ex vivo *complement activation assay*

To maintain complement activity, the blood samples were anticoagulated with lepirudin (Refludan, Schering, Berlin, Germany). Increasing concentrations of S(wt)F(wt) or S(wt) (0–2000 nM) were incubated in full blood for 30 min at 37°C under continuous rotation (300 rpm). Complement activity was stopped by adding 10 mM EDTA in the suspension, and the plasma was separated from the blood cells by centrifugation at 5000 *g*. A 1:30 dilution of each plasma sample was analyzed by the MicroVue SC5b-9 Plus Enzyme Immunoassay, according to the manufacturer’s instructions (Quidel, San Diego, CA, USA). One *S. aureus* colony (1 × 10^8^ cells) was used as a positive control for SC5b-9 formation. The bacteria were grown overnight on a blood agar plate at 37°C 5% CO_2_ atmosphere.

#### Fluorescence microscopy

Cells were imaged using a Nikon A1R/Storm microscope using a ×100 numerical aperture oil-immersion Nikon TIRF objective lens. We used a TIRF microscopy module and laser excitation at wavelengths 488 nm (for mGFP), 561 nm (for Alexa Fluor 594), and 647 nm (for Alexa Fluor 647) from a commercial multilaser unit fiber coupled into the microscope, capable of delivering maximum power outputs up to ∼200 mW with a depth of penetration in the range of ∼100–130 nm for the TIRF excitation evanescent field. Fluorescent images were acquired on an iXon+ 512 electron-multiplying charge-coupled device camera detector (Andor, Belfast, Northern Ireland) at a magnification of 150 nm/pixel. Green and red channel images were obtained by imaging through separate GFP or Alexa Fluor 647 filter sets. For high laser excitation intensity single-molecule millisecond imaging, green channel images to determine mGFP localization were acquired continuously using 488 nm wavelength laser excitation over a period of ∼5 min through a GFP filter set; then, the filter set was manually switched to Alexa Fluor 647 for red channel image acquisition continuously using 647 nm wavelength laser excitation until complete photobleaching of the sample after 1–2 min. For photobleaching, laser powers ranged between 15 mW (Alexa Fluor 647) and 100 mW (mGFP). For fixed cell analysis, cells were either incubated first with mS or mS*, washed and incubated with mF or mF*, or incubated first just with mS* with mF* absent and then washed and fixed with 1% paraformaldehyde.

For fluorescence imaging, the HEK cells were grown on 0.1% poly-l-lysine, which coated 8-well-chambered cover-glass slides (Ibidi, Martinsried, Germany) in standard growth conditions described above. To analyze the deposition of mS* on live cells, the cells were first imaged in PBS buffer in the absence of toxin. Here, a 256 × 256 pixel area covered ∼1 cell per field of view. Then, the cells were incubated for 2 min with 5 µg/ml Alexa Fluor 594 maleimide mS* in RPMI-HSA, and the cells were carefully washed with PBS, keeping the imaging area and focus constant. Because of the fast bleaching of the Alexa Fluor 647 label, a more stable Alexa Fluor 594 label was used for the mS* deposition imaging. The deposition of mS* was detected for 10 min, and the lysis of the cell was recorded for 15 min after addition of 600 nM unlabeled mF. Cells were imaged in TIRF at 50 ms per frame with the laser automatically switched among 488 nm/0.22 mW, 647 nm/3 mW, and 561 nm/3 mW or 488 nm/0.22 mW and 561 nm/3 mW.

#### FRET experiments

The Cy3 mS* or nonlabeled mS, Cy3 mF* or nonlabeled mF, or Cy3 mF* and controls (only FITC-labeled cells or unlabeled cells with only Cy3 mS*) were incubated at 4°C for 30 min and then 10 min at 37°C, washed 2 times with RPMI-HSA, and fixed as before. The cells were in PBS during imaging. FITC experiments were performed using a Leica TCS SP5 microscope, a 62 times oil-immersion objective lens, and FRET Sensitized Emission Wizard in Leica Application Suite Advanced Fluorescence. Images were acquired using 488 and 543 nm wavelength lasers, a laser power of 27% 12.0 (A.U.), and a scan size of 512 × 512, 800 ms, 50 ms per frame, beam splitter triple dichroic 488/543/633.

FRET efficiency ε was calculated using the donor, directly excited acceptor, and donor excited acceptor intensity from *n* = 5–10 manual regions of interest inside cells for each experiment, using the following formula (37):
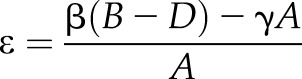
where *B* = intensity signal, donor excited acceptor; *D* = intensity signal, donor; *A* = intensity signal, directly excited acceptor; β = calibration for ratio of measured intensities of *B*_donor channel_/*A*_donor channel_; and γ = calibration for ratio of measured intensities of *B*_acceptor channel_/*A*_acceptor channel_.

#### Single-molecule imaging of live and fixed cells

GFP and Alexa Fluor 647 fluorescence micrograph time series of fixed and live cells were sampled taken at 20 ms per frame. Green channel images were acquired continuously using 488 nm wavelength laser excitation over a period of ∼5 min *via* the GFP filter set. Then, the filter set was manually switched to that for Alexa Fluor 647, and red channel images were acquired continuously using 647-nm wavelength laser excitation until complete photobleaching of the sample after 1–2 min. The step-wise, single-molecule fluorescence photobleaching was analyzed both for live and fixed cells. For live cell photobleaching analysis, the cells were incubated with 150 nM mS* or unlabeled mS, as required for ∼15 min. After washing with PBS, 600 nM mF* or unlabeled mF was added, and the imaging was done immediately within 10–15 min. If mF* was added, then the wells were washed with PBS before analysis. Furthermore, samples with only mS and without toxins were analyzed. For fixed cell analysis, the cells were incubated first with mS or mS* for 30 min at +4°C in RPMI-HSA, washed with the same buffer, and incubated for 10 min at 37°C with mF or mF*, or the same protocol was followed but using mS* alone with mF* absent. Then, the cells were washed and fixed with 1% paraformaldehyde. One molar mercaptoethylamine buffer was used for fixed cell analysis. Photobleaching of recombinant mGFP and Alexa Fluor 647 mS* was also separately analyzed in a tunnel slide comprising 2 pieces of double-sided tape, forming a channel sandwiched between a standard glass microscope slide and a plasma cleaned coverslip. mGFP or Alexa Fluor 647 mS* Solutions (1 μg/ml) were immobilized onto the coverslip coated by anti-GFP or anti-His antibodies, with PBS washes in between.

### Quantification and statistical analysis

#### Binding and permeability assays

Statistical significance between repeated (*n* > 1) experiments was analyzed using 2-tailed Student’s *t* tests, where use of a standard *P* < 0.05 threshold indicated statistical significance. Means ± sd of repeated experiments are shown in error bars, unless indicated otherwise.

#### Image analysis

Basic image extraction, cropping, and quantification were done using NIS-Elements microscope imaging software and ImageJ (U. S. National Institutes of Health, Bethesda, MD, USA; http://rsb.info.nih.gov/ij/). More advanced focus tracking was done using bespoke software written in MatLab (MathWorks, Natick, MA, USA) (38), which enabled automatic detection and localization of individual fluorescent foci to within 40 nm lateral precision (Supplemental Fig. S4). The software identifies candidate foci by a combination of pixel-intensity thresholding and image transformation. The intensity centroid and characteristic intensity, defined as the sum of the pixel intensities inside a 5-pixel radius circular region of interest around the focus intensity centroid minus the local background and corrected for nonuniformity in the excitation field, are determined by repeated gaussian masking. If the signal-to-noise ratio of a focus (the intensity per pixel/background sd per pixel) is greater than a preset threshold, nominally here set at 0.4 based on prior simulations, then it is accepted and fitted with a 2-dimensional radial gaussian function to determine its width. Foci in consecutive frames within a single point spread function (PSF) width, and not different in intensity or width by greater than a factor of 2, are linked into the same track.

Focus intensity was used to quantify stoichiometry information. As foci photobleach over time during continuous laser excitation, their intensity falls in a stepwise manner as a result of photobleaching of an integer number of fluorophore tags in each sampling time window. With the quantification of the size of a single step, the characteristic intensity of a single fluorophore can be obtained and thus, the stoichiometry of the focus from its initial intensity. The step size is found from the periodicity in the distribution of focus intensities corroborated by the pairwise distance distribution of these intensities and the Fourier spectrum of the pairwise distance that contains peaks at the characteristic intensity and harmonics at multiples of this value (Supplemental Fig. S4*D, E*).

Here, the copy number of hC5aR-mGFP was comparatively high, such that the TIRF images were initially saturated regarding pixel-intensity output. After ∼20 s of photobleaching, the nonsaturated focus intensity values were fitted by an exponential function that characterized the rate of intensity decay, equivalent to an exponential photobleach time of ∼20 s, and extrapolated back to 0 time to determine the initial focus intensity (Supplemental Fig. S4*F*). The Alexa Fluor 647 dye also bleached during 647 nm wavelength laser excitation, but images were not initially saturated. Some images, which were exposed to the 488 nm laser and then the 647-nm laser, were also bleached by the 488-nm wavelength laser. In these images, a fixed correction factor of 6 times, determined by comparing with images exposed to the 647-nm laser first, was used. The stoichiometry of each focus was then determined as the initial intensity divided by the intensity of the appropriate single fluorescent dye tag (*i.e.*, either mGFP or Alexa Fluor 647 in this case).

We characterized the mobility of tracked foci by calculating their mean squared displacement (MSD) as a function of time interval (τ). For each detected focus, the MSD was calculated from the measured intensity centroid [*x*(*t*),*y*(*t*)] at time *t*, assuming a focus track of *N* consecutive image frames at a time interval τ = *n*Δ*t*, where *n* is a positive integer, and Δ*t* is the frame integration time (here, 20 ms):
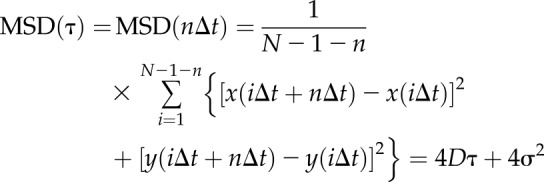
The lateral (*xy*) localization precision is given by σ, which we determine to be 40 nm. We fitted a straight line to each separate MSD relation. Assuming a line fit has an optimized gradient *g* to the first 4 points (defined as the first 3 measured MSD data points for *n* = 1, 2, and 3, in addition to a fourth data corresponding to *n* = 0, obtained from constraining the intercept 4σ^2^ to within measurement error of the localization precision), then the estimated microscopic diffusion coefficient *D* is *g*/4.Δ*t*. For immobile foci, tracks were collated and compiled to generate a mean MSD *vs.* τ relation, which was fitted to an asymptotic rising exponential function as an analytical model for confined diffusion of MSD plateau equal to *L*^2^/6, where *L* is the effective confinement diameter (39), enabling us to estimate the confinement diameter.

#### Colocalization analysis

The structural similarity index (SSI) was calculated on intensity-normalized images using the in-built MatLab function based on Wang *et al.* (40). This index uses a combined luminance, contrast, and structural term, based on local means ± sd and cross-correlation to assess the similarity of images.

The extent of colocalization between red and green detected foci was determined using a method that calculated the overlap integral between each green and red focus pair whose centroids were within ∼1 PSF width (∼3 pixels). With the assumption that 2 normalized 2-dimensional gaussian intensity distributions *g*_1_ and *g*_2_ for green and red foci, respectively, centered around 
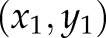
 with σ width σ*_1_* and around 
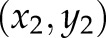
 with width σ_2_, the overlap integral *ν* is analytically determined as:
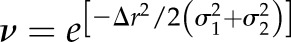
where:
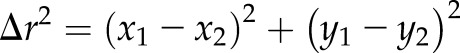
We use a criterion of an overlap integral of 0.75 or above to indicate putative colocalization (41), as this corresponds to a focus centroid separation equivalent to the localization precision in this case. By quantification of the sd on the number of detected foci in each channel, we estimate that the se of colocalization proportion under our typical imaging conditions is ∼9%.

#### Random focus overlap models

We calculated the probability of focus overlap in a single color channel by first estimating a sensible range of focus surface density *n*. For the lower limit, we used the number of focus tracks detected in a 20 image frame time window, and for the upper limit, we used the average measured value of the background-corrected pixel intensity value divided by the intensity of a single fluorophore (equivalent to ∼1 mS* molecule per pixel). We implemented these probability estimates into a surface density model that assumed a random Poisson distribution for nearest-neighbor separation (41–46). This model indicates that the probability that a nearest-neighbor separation is greater than *w* is given by exp(−π*w*^2^*n*). The probability of overlap for each density estimate (Supplemental Fig. S6) was convolved with a real molecular stoichiometry distribution and a gaussian function *p*(*x*) of stoichiometry (*x*)*:*
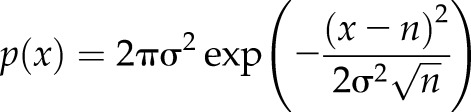
where σ is the width of single fluorophore intensity distribution (∼0.7 molecules), and *n* is the real molecular stoichiometry. The tetramer model assumes *n =* 4, and then all higher-order stoichiometries are a result of overlapping PSFs. The tetramer oligomer molecule assumed an equal number of multimerized tetramers up to 5, which gave the best fit to the data.

The same strategy was used to model the random overlap probability for green and red color channel fluorescent foci in dual color imaging experiments to assess the extent of apparent colocalization as a result of random overlap between hC5aR and mS*/mF*. The probability that a nearest-neighbor separation is greater than *w* for foci of 2 different types is the same as a single type multiplied by two-thirds (38).

### Software

All of our bespoke software developed is freely and openly accessible *via* the SourceForge website (*https://sourceforge.net/projects/york-biophysics/)* (47).

### Statistical tests and replicates

All statistical tests used are 2-tailed unless stated otherwise. For single-molecule TIRF imaging, each cell can be defined as a biologic replicate sampled from the cell population. We chose sample sizes of 5–7 cells yielding thousands of foci, generating reasonable estimates for stoichiometry and diffusion coefficient distributions. Technical replicates are not possible with the irreversible photobleaching assay.

## RESULTS

### Maleimide-labeled LukSF mediates toxicity on human PMN and HEK cells

**Figure 1:**
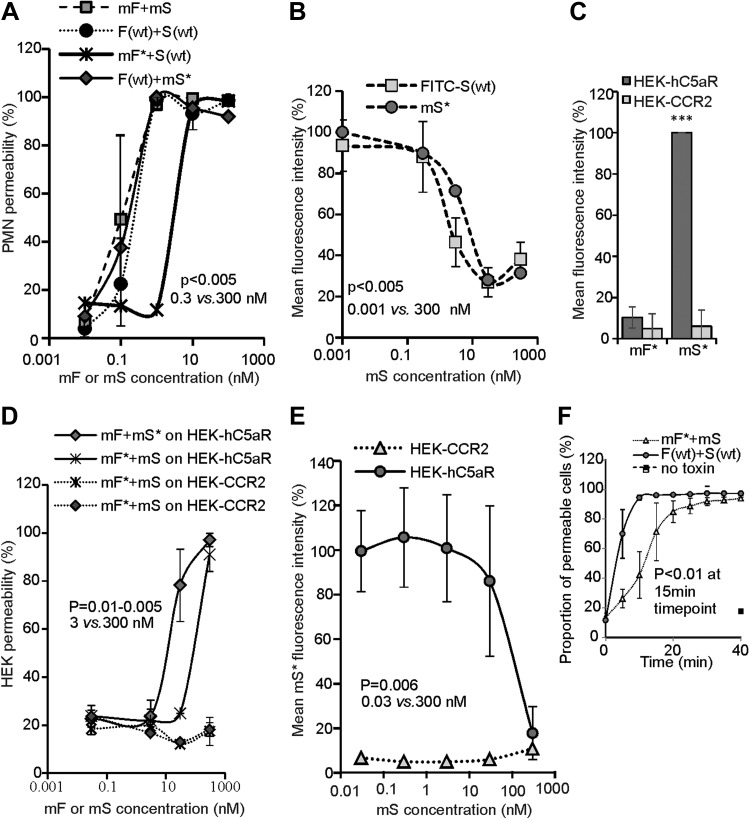
Toxin functionality on PMN and HEK cells. *A*) PMN cell permeability in the presence of unlabeled LukSK281CY113H and LukFK288C (mS + mF; number of biologic replicates, *n* = 2), Alexa Fluor mS* or mF*, and F(wt) or S(wt) (*n* = 1), compared with PMN cell permeability of S(wt) and F(wt) (*n* = 3). *B*) Inhibition of 3 µg/ml FITC-labeled FITC-S(wt) (*n* = 3) and mS* (*n* = 1) binding to PMN cell by mS. Permeability dose dependencies for *A* and *B* are shown with a polynomial spline fit; statistical significance indicated between low (0.3 and 0.001 nM) and high (300 nM) toxin concentrations using Student’s *t* test. Error bars indicate sd. *C*) Column indicating binding responses for mF* on hC5aR cells (*n* = 2). ***P < 0.001, statistically significant difference between mS* binding on HEK-hC5aR cells compared with HEK-CCR2 and mF* binding on these cells. *D*) Permeability of hC5aR-transfected HEK cells using unlabeled mS and mF and Alexa Fluor mS* and mF* compared with S(wt) and F(wt) (*n* = 2). *E*) Inhibition of 3 µg/ml mS* binding by mS on HEK-hC5aR cells (*n* = 3). CCR2-transfected HEK cells used as negative controls for toxin binding and lysis in *C* and *D* (*n* = 2) or 1 representative experiment in *C*. Dose dependency shown with polynomial spline fit. Statistical significance calculated between low (0.3 and 0.001 nM) and high (300 nM) toxin concentrations using Student’s *t* test. *F*) Permeability response of hC5aR-transfected HEK cells following incubation with unlabeled mS and Alexa Fluor maleimide mF* or toxins F(wt) and S(wt) (*n* = 3). Statistical significance calculated between 15 and 0 min time points using Student’s *t* test. Error bars indicate sd. Percentages of mean fluorescence intensity are shown as relative to the maximum intensity in each individual experiment (*B*, *C*, *E*). Permeability of the cells was analyzed after 30 min incubation at +37°C, whereas the inhibition assays were analyzed after 45 min incubation at +4°C (Supplemental Movie S3).

To study LukSF pore formation on live cells using single-molecule fluorescence microscopy, single cysteine substitutions on the exposed surface of the cap domain of the individual toxins (Supplemental Fig. S1*B*)—K288C on LukF and K281C on LukS—were engineered to facilitate maleimide labeling. These were denoted as the modified protein mF or mS. A second substitution Y113H on LukS was chosen on the stem domain to facilitate pore formation of the LukS mutant (mS), based on previous studies (17). We compared the lytic activity of these mutants with their unmodified wild-type equivalents by measuring PMN membrane permeabilization after 30 min toxin exposure using the DNA-binding fluorescent dye DAPI by flow cytometry. DAPI does not penetrate intact cell membranes and is therefore a good measure for cell permeability and cell death. In this assay, each of the wild-type toxins was replaced with the modified protein either unlabeled (mF or mS) or with a single Alexa Fluor 647 dye molecule label (mF* or mS*; **Fig. 1**). All modified toxins induced PMN permeabilization, reaching 100% at ∼3 nM (Fig. 1*A*), interchangeable with the wild-type equivalents. Only maleimide-labeled LukF (mLukF) (mF*) lost activity and required ∼30 nM to reach 100% permeabilization. As the LukS component mediates the toxin recognition on the target C5aR, we evaluated the binding potency of mS and mS* on PMNs. In this assay, mS was able to inhibit the interaction of FITC-labeled S(wt) on PMNs equally as well as the maleimide mS* (Fig. 1*B*).

The LukSF toxin is known to be specific toward human cells expressing hC5aR, such as neutrophils, monocytes, and macrophages, but does not lyse cells that do not express the receptor (18). To report on the spatiotemporal localization of the receptor and for determination of the subunit stoichiometry in any observed receptor clusters, we prepared HEK cells expressing hC5aR with an mGFP variant (bearing the obligate-monomer mutation A206K) cloned in the C-terminal end of the receptor. This cell line also forms a monolayer on the coverslip and can be used for the introduction of a single dye on the cloned receptor, requirements for TIRF, and single-molecule imaging. We verified the specificity and activity of the mutated and labeled toxins on the HEK-hC5aR cells. As expected, the toxins lysed only cells expressing hC5aR, whereas control hCCR2-expressing cells, which do not bind LukS (48), remained intact. We did not observe any binding of mF* on the same cells (Fig. 1*C, D*), which is consistent with previous observations that LukF, in the absence of LukS, does not interact with PMN (16). The unlabeled mS inhibited binding of mS* in a dose-dependent fashion (Fig. 1*E*). HEK cells transfected with hC5aR required higher toxin concentrations for optimal binding and lysis by mS + mF compared with PMN, which is in agreement with previous data for the wild-type variants. An explanation for this finding could be that PMNs also express another ligand for LukS, C5LR (or C5aR2), and LukF (48, 49). It is known that C5aR expression levels are not stable in neutrophils but can easily change in natural settings, for example, as a response to increased C5a levels (50). We found that the expression levels between unstimulated PMNs and THP-1 monocytes were low (mean fluorescence intensity = 748 ± 29 and 1322 ± 74) and clearly different from phorbol 12-myristate 13-acetate-stimulated THP-1 cells (mean fluorescence intensity = 3167 ± 360) that are known to be sensitive to LukSF pore formation (18). Therefore, we examined HEK cells as a model cell to study hC5aR-LukSF, although their relative receptor expression levels (mean fluorescence intensity = 20,155 ± 1570) were high with slight overlap with the expression levels of activated cells (Supplemental Fig. S2). As hC5aR is expressed at low levels in unactivated neutrophils, we used this cell line as a model to study Luk–receptor interactions in higher resolution and to exclude the effect of the LukS–hC5aR2 interaction with the cells.

To be able to analyze the dynamics of receptor and toxin interactions, we verified the conditions required for HEK cell lysis in time in the presence of mF and mS. As the maleimide mF* required higher concentrations for efficient lysis of HEK cells and because of the loss of molecules during washing cycles, the assay was optimized to have 20-fold excess of mF* (600 nM). Following preincubation of hC5aR-mGFP-expressing HEK cells with S(wt) or mS, F(wt) or mF* was added, and the cellular uptake of DAPI was measured by flow cytometry every 5 min. The toxins F(wt) and S(wt) caused >80% cell toxicity within 10 min, whereas closer to 20 min was required for significant lysis by the mF* and mS toxin combination (Fig. 1*F*).

**Figure 2:**
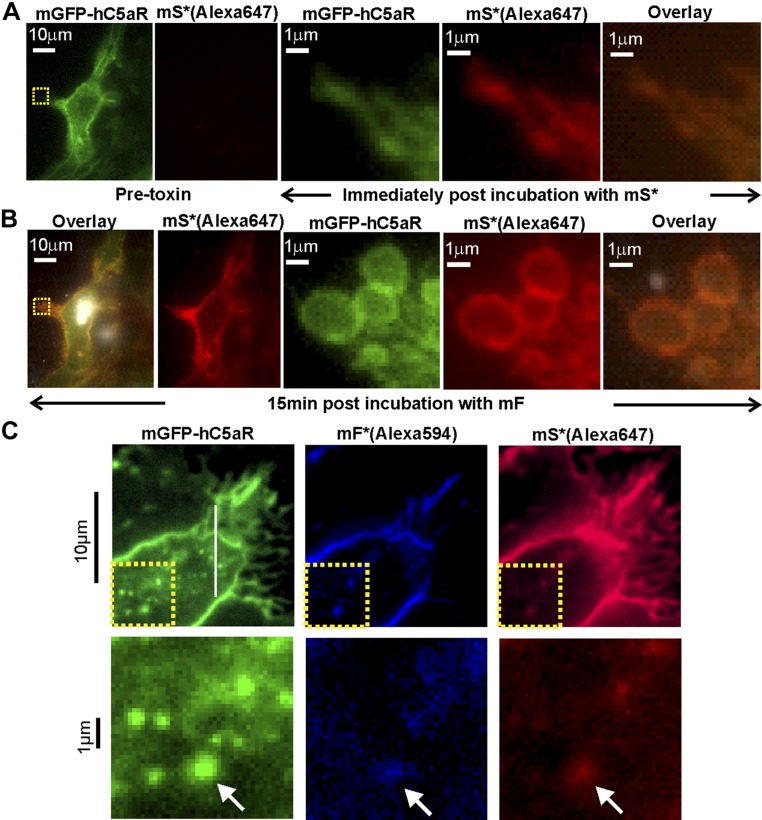
Standard TIRF microscopy of LukS/F with hC5aR on HEK cells. *A*, left) TIRF image of hC5aR-mGFP on the surface of a HEK cell before addition of toxin; *A*, right) zoom-ins of yellow, dashed square (left) immediately following 2 min incubation with Alexa Fluor 647-labeled LukSK281CY113H [mS*(Alexa647)]. *B*) Equivalent images of the same cell of *B* after >15 min incubation with LukFK288C (mF). *C*, upper) TIRF image of colocalization of Alexa Fluor 594- and Alexa Fluor 647 mF* and mS* [mF*(Alexa594) and mS*(Alexa647)] with hC5aR-mGFP on HEK cells; *C*, lower) zoom-in of yellow, dashed square (upper) with colocalized foci indicated (arrows).

We further confirmed cell lysis using our modified toxins and receptor by live cell imaging. We first set the conditions to facilitate data acquisition of dynamic events involved in the formation of LukSF nanopores in hC5aR-mGFP HEK cell membranes. We sampled every 2.5 s at 50 ms exposure time per frame using standard (nonsingle molecule) TIRF microscopy at very low excitation intensity to prevent photobleaching. Cells were first imaged in the absence of toxin. In the green channel, we observed mGFP localization consistent with the cell membrane manifest as relatively high apparent brightness toward the cell boundaries consistent with the cell membrane curving away from the microscope coverslip perpendicular to the TIRF excitation field. Controlled addition of mS* (labeled with Alexa Fluor 647) to the sample Petri dish, followed by washing, while imaging simultaneously throughout, resulted in similar localization of the hC5aR and mS* (**Fig. 2** and Supplemental Movie S1). We quantified this in images by calculating the SSI from intensity normalized regions of interest at the cell periphery and center (Supplemental Fig. S3) as a function of time. The SSI increased rapidly at the periphery and more slowly in the center, as the edges are more accessible to mS*. Further addition of mF resulted in complete lysis of the cell, as defined by the observation of explosive release of membrane vesicles, after ∼15 min (Fig. 2 and Supplemental Movie S2) and a decrease in the SSI at the periphery (Supplemental Fig. S3*B*). We also observed hC5aR-GFP, mS* (Alexa647), and mF* (Alexa594) together in 3 color experiments, imaging cells after addition of toxins and washes until the start of lysis (Fig. 2). Similar localization was indicated by the high SSI among all 3 channels (Supplemental Fig. S3*C* and Supplemental Movie S3).

### Single-molecule TIRF microscopy of live cells indicates that the clustered C5aR binds LukS with a tetrameric substructure

**Figure 3:**
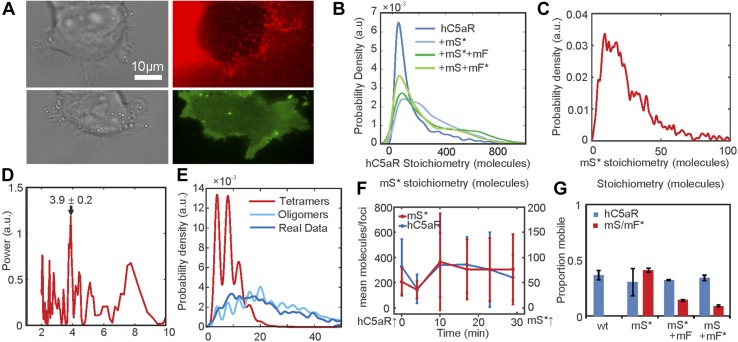
Single-molecule TIRF microscopy of hC5aR, LukS, and LukF in live cells. *A*) Images of HEK cells treated with LukSK281CY113H (mS) and Alexa Fluor-labeled LukFK288C (mF*) showing brightfield (left), hC5aR-mGFP (green), and mF* (red). *B*–*D*) Probability distribution for stoichiometry of hC5aR in the absence and presence of Alexa Fluor mS* and mF* (*B*), and of mS* foci (*C*), indicating tetramer periodicity (*D*) from Fourier spectral analysis. *E*) A random tetramer overlap model cannot account for mS* experimental stoichiometry data (*R*^2^ < 0), but a tetramer-multimer model results in excellent agreement (*R*^2^ = 0.85). *F*) hC5aR and mS* stoichiometry as a function of incubation time. *G*) Proportion of immobile and mobile colocalized foci in the presence and absence of mS and mF. Error bars show sem from *n* = 5–15 image subregions (*n* = 20–30 cells,∼1000–10,000 foci).

With the use of higher laser intensity, TIRF excitation enabled rapid millisecond single color channel sampling of single fluorophores faster than their molecular mobility in the cell membrane (51), confirmed by imaging antibody-immobilized mGFP and Alexa Fluor dyes (Supplemental Fig. S4). Imaging live hC5aR-mGFP cells in these conditions saturated the camera charge-coupled device, but after 1–2 min of exposure, photobleaching was sufficient to reduce intensity and allow us to observe several distinct, mobile, circular fluorescent foci at a mean surface density of ∼1/µm^2^ in the membrane regions that lie parallel to the TIRF field away from the cell boundaries (**Fig. 3*A*** and Supplemental Movie S4). We monitored the spatiotemporal dynamics of foci in the planar membrane regions using automated tracking software (38), which allowed foci to be tracked for up to 18 s to a spatial precision of ∼40 nm (52) below the diffraction limit, thus enabling super-resolution localization data to be obtained. The measured width of the focal waist (defined as the half-width at half-maximum, determined from their pixel-intensity profile) was in the range of 200–300 nm, consistent with the PSF width of our microscope (Supplemental Fig. S4*G*). By use of a step-wise photobleaching analysis, we estimated stoichiometry values for all detected fluorescent foci by using a method that quantifies the initial unbleached focus brightness and divides this by the measured brightness for the relevant single-dye reporter molecule (Supplemental Fig. S4) (53). These foci contained large numbers of receptors with a mean stoichiometry of ∼180 (Fig. 3*B* and **Table 2**). Addition of mS and mF increased the mean stoichiometry by >50%, consistent with the toxin causing receptor clustering.

**TABLE 2:** Stoichiometry and microscopic diffusion coefficient in live and fixed cells

Type	Stoichiometry, mol (mean ± sd)	Foci (*n*)	Mobile foci *D*, μ^2^m/s (mean ± sd)	Immobile foci *D*, μ^2^m/s (mean ± sd)
Live cells				
C5a	185 ± 224	5346	0.48 ± 0.43	0.03 ± 0.03
C5a + mLukS*	281 ± 213	8272	0.49 ± 0.42	0.02 ± 0.03
C5a + mLukS* + mLukF	291 ± 248	6605	0.46 ± 0.42	0.03 ± 0.03
C5a + mLukS + mLukF*	223 ± 202	4981	0.47 ± 0.40	0.02 ± 0.03
mLukS*	29 ± 22	999	0.44 ± 0.45	0.03 ± 0.03
mLukS* + mLukF	84 ± 89	841	0.34 ± 0.35	0.01 ± 0.02
mLukS + mLukF*	6 ± 4	557	0.40 ± 0.44	0.02 ± 0.03

	Colocalized C5a foci, *n* (/%)	C5a foci not colocalized, *n* (%)	Stoichiometry of colocalized C5a foci, mol (mean ± sd)	Stoichiometry of C5a foci that are not colocalized, mol (mean ± sd)
Fixed cells				
C5a + mLukS*	89 (32)	193 (68)	36 ± 26	35 ± 24
C5a + mLukS* + mLukF	84 (7)	1079 (93)	37 ± 25	32 ± 24
C5a + mLukS + mLukF*	59 (83)	283 (17)	26 ± 20	26 ± 20

	Colocalized Luk foci, *n* (%)	Luk foci not colocalized, *n* (%)	Stoichiometry of colocalized Luk foci, mol (mean ± sd)	Stoichiometry of Luk foci that are not colocalized, mol (mean ± sd)
mLukS*	88 (7)	1198 (93)	72 ± 47	61 ± 44
mLukS* + mLukF	86 (88)	658 (12)	121 ± 95	136 ± 94
mLukS + mLukF*	60 (5)	1186 (95)	20 ± 16	18 ± 15

The imaging of mS* incubated with hC5aR-mGFP cells revealed distinct foci (Fig. 3*A* and Supplemental Movie S5). The probability distribution of mS* stoichiometry values in live cells in the absence of mF is shown in Fig. 3*C*, rendered using a kernel density estimation that generates an objective distribution that does not depend on the size and location of subjective histogram bins (54). We measured a broad range of stoichiometry values, spanning a range from only a few mS* molecules per focus to several 10s of molecules, with a mean of ∼30 molecules per focus. Closer inspection of the stoichiometry values indicated an underlying periodicity to their probability distribution, which we investigated using Fourier spectral analysis (46). The resulting power spectrum (Fig. 3*D*) indicated a fundamental peak equivalent to a stoichiometry of 3.9 ± 0.2 molecules, suggesting that foci are composed of multiples of tetrameric mS* complexes with hC5aR.

Fluorescent foci, if separated by less than the diffraction-limited PSF width of our microscope, are detected as a single particle but with higher apparent stoichiometry. Therefore, we tested the hypothesis that the observed mS* focus stoichiometry distribution could be explained by the random overlap of isolated mS* tetramer foci. To do so, we modeled the nearest-neighbor separations of individual mS* tetramers in the cell membrane as a random Poisson distribution (41) and used sensible ranges of tetramer surface density based on our single-particle tracking results (Supplemental Fig. S5). However, all random tetramer overlap models that we explored showed poor agreement to the observed experimental stoichiometry distribution, but we found that random overlap of multimers of tetramers could account for the stoichiometry distribution well (Fig. 3*E*). Optimized fits indicated that the random overlap of mS* foci with a stoichiometry in the range of 4–20 molecules was able to account best for the experimental data. As hC5aR is clustered, this likely accounts for the clustering of mS* but not its tetrameric periodicity. These results are consistent with mS* binding to clusters of hC5aR with a bias toward tetrameric substructures.

We tested if there was a dependence of focus stoichiometry on incubation time with Luk. The acquirement of a time course for mF* accumulation following preincubation of cells with mS was not feasible, as unbound mF* had to be washed from the sample to prevent a prohibitively high fluorescent background. However, we were able to acquire time courses in which mF was added to cells that had been preincubated with mS*. For these, the mS* focus stoichiometry distribution was measured as a function of time after mF addition for several different fields of view, each containing typically ∼5 cells. We found that the mean hC5aR focus stoichiometry indicated no obvious correlation to mF incubation time (Fig. 3*F*); however, the mean mS* focus stoichiometry increased with time (*P* < 0.05).

With the calculation of the MSD as a function of time interval (τ) for each tracked focus and fitting a line to the first 4 points, we could determine its apparent microscopic diffusion coefficient (*D*). The distribution of *D* for hC5aR and mS*/mF (Supplemental Fig. S6) had similar low value peaks at ∼0.05 µm^2^/s, consistent with immobile foci tracked with our localization precision of 40 nm. Several mobile foci were also seen (Supplemental Movies S6 and S7), which diffused at rates up to ∼5 µm^2^/s. Based on the measured width of the immobile peak width on these distributions and informed by simulations of immobile foci with representative intensity and noise (Supplemental Fig. S6), we set a threshold of 0.12 µm^2^/s to categorize foci as either immobile, which indicated a mean *D* = 0.025 ± 0.030 µm^2^/s, or mobile, which indicated a mean *D* = 0.47 ± 0.40 µm^2^/s (Table 2). Plots of the measured MSD *vs.* τ relations for mobile foci indicated a linear dependence indicative of free Brownian (*i.e.*, normal) diffusion, with no measureable differences between the receptor alone and the receptor bound to toxin. We also noted, qualitatively, a broad trend for a decrease in diffusion coefficient with an increase in receptor stoichiometry. Similar plots for immobile foci indicated a more asymptotic dependence consistent with confined diffusion (39) whose plateau was equivalent to a confinement diameter of ∼400 nm (Supplemental Fig. S6) but again, with no measureable difference between the bound and unbound receptor populations. The relative proportion of mobile foci was ∼35% of tracked foci for hC5aR, regardless of toxin, and similar for mS in the absence of mF. Addition of mF, however, caused a drop in the mobile proportion of toxin by a factor of ∼3 (Fig. 3*G*), suggesting that LukF causes insertion of the complex and because only mF*/mS* were affected, a possible change in association of the LukSF complex with hC5aR. An alternative explanation could be that the reduction in mobility is because pores are sticking to newly immobile receptors correlated to toxin binding in some undetermined way; however, we believe this is a less plausible explanation, as we observe an insensitivity in the proportion of the mobile receptor population regarding addition of either toxin component.

### Single-molecule TIRF microscopy combined with colocalization analysis of fixed cells suggests that LukSF dissociates from the receptor

**Figure 4:**
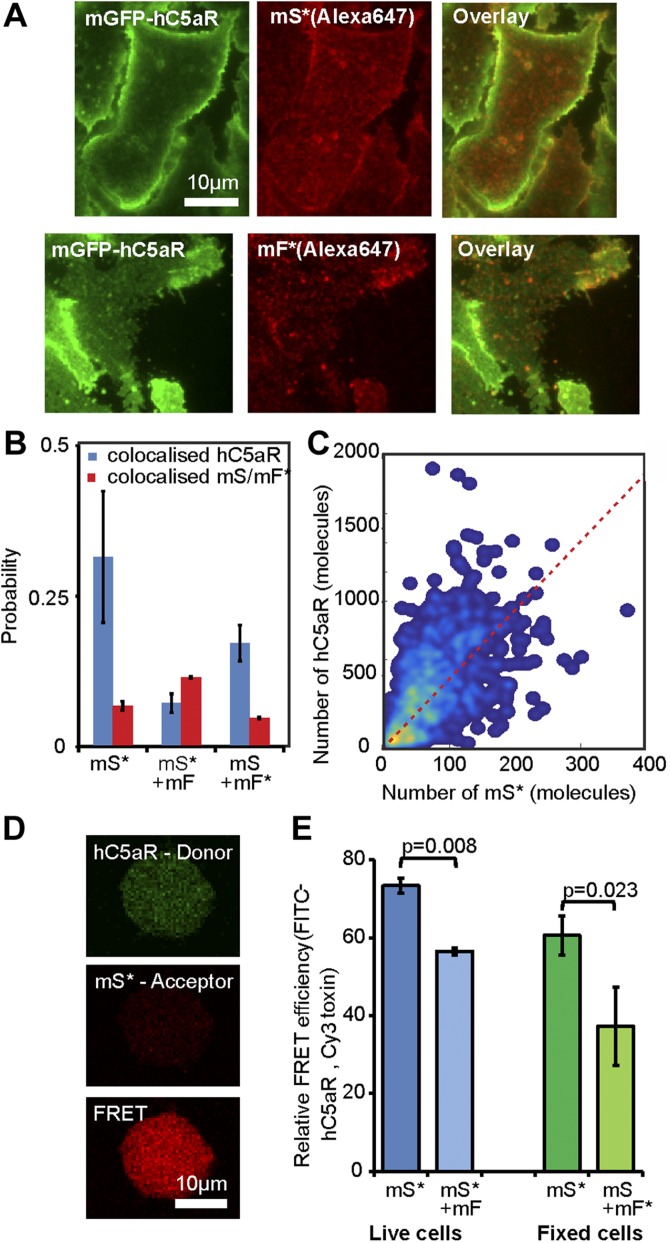
Relative stoichiometry of hC5aR, LukS, and LukF in fixed cells. *A*) Micrographs of fixed hC5aR-mGFP HEK cells treated with LukSK281CY113H (mS) and LukFK288C (mF) showing hC5aR-mGFP (left), Alexa Fluor 647 (Aöexa647, middle), and merge (right) on Alexa Fluor mS* with zoom-in (lower panels) showing colocalized foci. *B*) Proportion of colocalized foci treated with mS, mS* + mF, and mS + mF* for hC5aR. Error bars show sem from *n* = 4 image subregions (*n* ∼ 1000 foci). *C*) Heatmap of correlation between hC5aR and mS stoichiometry (red, dashed line indicates 4 mS per hC5aR molecule); *R*^2^ ∼ 0.15 (*n* ∼ 1000 foci from ∼ 10 cells). *D*, *E*) FRET images and efficiencies. The FRET experiment was performed in live and fixed sortase-tagged FITC-hC5aR-expressing cells. Live cells (number of biologic replicates, *n* = 2) were incubated in the presence of Cy3 mS* for 1 h at +4°C and washed, after which, unlabeled mF was added. FRET was analyzed before (mS*) or after (mS* + mF) addition of mF. FRET from fixed cells (*n* = 3) was analyzed in the presence of mS* or unlabeled mS and Cy3 mF* (*n* = 2). Statistical significance between cells with only mS and both of the toxin components, mS and mF, was analyzed using Student’s *t* test. Error bars indicate sd.

As a result of the high image frame rate of single-molecule TIRF microscopy, we were not able to image simultaneously 2 color channels on our microscope; rather, each channel was imaged separately in the same cells. Therefore, to determine whether the toxin remains bound to the receptor and to quantify the relative stoichiometry of components, we imaged fixed cells, halting cell lysis, using the same two spectrally distinct green/red dyes of mGFP and Alexa Fluor 647 to label receptor and toxin components, respectively, as for the live cell experiments. We imaged cells incubated with mS*, followed by incubation with mF (**Fig. 4*A***), as well as simultaneously with mS + mF* (Supplemental Fig. S7), and observed foci with similar stoichiometries (Table 2) to live cells but colocalized with hC5aR. The SSI values were lower for these fixed cells (Supplemental Fig. S7) compared with live cells. Approximately 32% of the hC5aR foci were found colocalized in the presence of mS*, dropping to <10% in the presence of mF (Fig. 4*B*). This low percentage was within our estimate of the degree of random colocalization between the green and red fluorophores, entirely down to chance, of ∼10%. This suggests that in the presence of mF, the toxin is not colocalized with the receptor and that mF causes disassociation from hC5aR. The SSI values also dropped in the presence of mF (Supplemental Fig. S8), consistent with this hypothesis. The stoichiometry values for detected green hC5aR-mGFP foci were calculated and plotted against the equivalent stoichiometry estimates for colocalized red foci of mS* and mF*, respectively (Fig. 4*C* and Supplemental Fig. S7) In the presence of mS* but in the absence of mF, the hC5aR-mGFP focus stoichiometry showed an approximately linear dependence on number of associated mS* molecules, suggesting that each colocalized mS* molecule was associated, on average, with ∼4–5 hC5aR molecules. In the presence of mF* or unlabeled mF, no dependence was observed (Supplemental Fig. S7; *R*^2^ < 0) consistent with a random association between toxin and receptor. These results are unlikely to be a result of fluorescence quenching, as it would need to be near 100% quenching to detect no Alexa Fluor fluorescence in the hC5aR-mGFP foci, and the drop in colocalization is observed independent of the labeled toxin used, either mS* with mF or mF* with mS.

### Live whole-cell FRET and biochemical measurements also support LukSF disassociation

We performed FRET experiments on FITC sortase-labeled hC5aR and Cy3 mS* or mF*, as donor and acceptor, respectively, in live cells to probe further the association between toxin and receptor. A FRET signal from whole cells of 75% efficiency was observed, with a statistically significant drop (*P* = 0.008, Student’s *t* test) to 56% when incubated with unlabeled mF (Fig. 4*D, E*), as would be expected if the complex formation leads to dissociation of the toxin from the receptor. To examine possible FRET between hC5aR and Cy3 mF*, we performed similar experiments on fixed cells. In these experiments, a FRET efficiency of 60% was observed between hC5aR and mS*, dropping below 40% (*P* = 0.023, Student’s *t* test) between hC5aR and mF*. As expected, no FRET signal was observed in the negative control, where only Cy3 mF* was present. These results are also consistent with the finding that hC5aR dissociates from the LukSF pore, although conformational or local environment changes cannot be ruled out with FRET alone, as the relatively high remaining signal might, in principle, also indicate remaining association or other inter- or intra-hC5aR–Luk interaction. The greater drop in FRET when measured with mF compared with mS might be caused by the 3–4 nm further distance of LukF from hC5aR.

**Figure 5:**
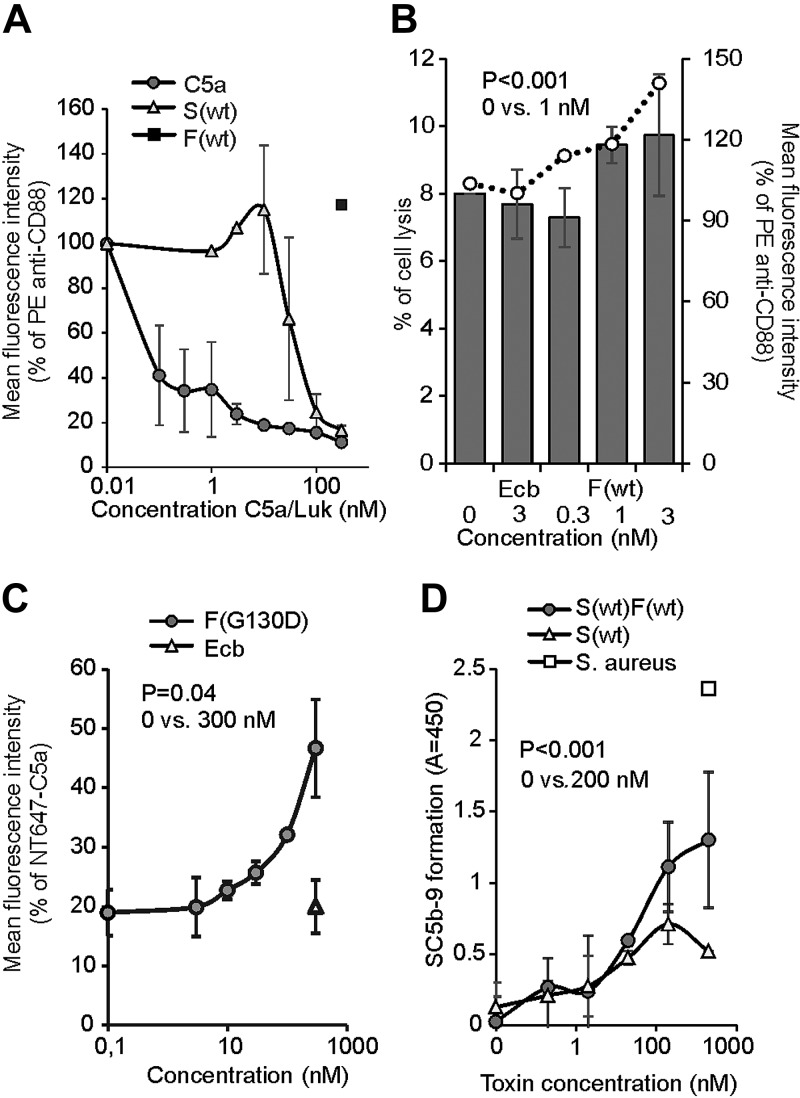
LukSF dissociation and rebinding of C5a on hC5aR-expressing cells. *A*) Inhibition of anti-CD88 binding on hC5aR-expressing HEK cells using increasing concentrations (*x* axis) of S(wt) and C5a; F(wt) is used as a negative control for inhibition of anti-CD88 binding (number of biologic repeats, *n* = 2). The values are normalized against the maximum binding observed with only anti-CD88. *B*) Disengagement of hC5aR from LukSF was observed as an increase in PE-conjugated anti-CD88 binding (right *y* axis, indicated with bars) on S(wt)-precoated cells using increasing but sublytic concentrations (*x* axis, indicated with dots) of F(wt). The values are normalized against the maximum binding observed with S(wt)-incubated cells with only anti-CD88. Minimal cell lysis (percent of lysed cells, left *y* axis) detected in F(wt) concentrations below 3 nM (*n* = 3). *C*) Rebinding of constant amount of NT647-labeled C5a on hC5aR upon LukSF formation, analyzed by incubating S(wt)-precoated cells with increasing concentrations (*x* axis) of LukF mutant F(G130D) that associates with LukS but does not lead to cell lysis (*n* = 2). The values are normalized against the maximum binding observed with only NT647-C5a. *D*) Effect of LukSF-mediated cell lysis on complement activation and C5a formation on full blood measured by using C5b-9 as a marker for complement activation in plasma (*n* = 3). Maximal C5a formation is observed by the incubation of full blood with live *S. aureus* bacteria. Ecb (*B*, *C*), F(wt) (*A*), or S(wt) (*D*) is used as a negative control in the assays. Percentages of mean fluorescence intensities is shown as relative to the maximal intensity in each individual experiment (*A*–*C*). Statistical significances are calculated using Student’s *t* test. Error bars indicate sd.

To confirm further that the LukSF complex dissociates from the target receptor, we used a PE-labeled anti-CD88 mAb to detect the liberation of free hC5aR receptors on the cell membrane upon LukSF formation. We first confirmed the ability of both C5a and S(wt) to compete for binding of the anti-CD88 antibody to the hC5aR-expressing HEK cells. Both ligands showed clear inhibition of anti-CD88 binding at 100 nM concentrations, whereas F(wt) was ineffective (**Fig. 5*A***). However, when the hC5aR-expressing cells were incubated with 100 nM S(wt), followed by incubation with increasing concentrations of F(wt) to form an active toxin, a statistically significant increase in anti-CD88 binding was detected at a F(wt) concentration of 1 nM when compared with no F(wt) (0 nM). Addition of a control protein Ecb did not change anti-CD88 binding. Cell permeabilization was measured in parallel and proved to be “sublytic,” enabling proper detection of liberated anti-CD88 without significant cell lysis (percent of lysed cells <10%; Fig. 5*B*). At a 3 nM F(wt) concentration, the proportion of dead cells increased above 10%, which determined the maximum concentration and increase in anti-CD88 binding that could be measured. The changes in C5aR mobility, colocalization, and FRET, with addition of LukF, combined with the biochemical evidence of anti-CD88 rebinding on hC5aR upon F(wt) addition on S(wt)-coated cells, are strongly indicative of disassociation of the LukSF complex.

### Dissociation of LukSF pores from hC5aR allows rebinding of C5a

As C5a is the natural ligand for hC5aR and can outcompete binding of LukS on the receptor, we next analyzed whether LukSF formation and disengagement of hC5aR would allow rebinding of C5a on the receptor. We specifically chose to analyze binding of labeled C5a and not LukS in the presence of increasing concentrations of LukF. This is because the addition of labeled LukS to cells coated with LukS (and then washing), together with LukF, will give several possibilities for association: for example, new pores for free or unoccupied C5aR, intercalation with present-bound LukS and LukF in pores, and binding to free or unoccupied C5aR but without pore formation. To detect C5a rebinding at higher LukF concentrations, we used a F(G130D) mutant that interacts with LukS but does not cause cell lysis. Nonlytic activity of this mutant in this assay was confirmed by DAPI staining that showed minimal cell lysis even at higher concentrations [9% at a 300 nM F(G130D) concentration]. At a 300 nM concentration, a significant increase in C5a binding was detected, indicating that LukSF dissociates from hC5aR, enabling simultaneous rebinding of an hC5aR-interacting ligand (Fig. 5*C*). On the contrary, addition of the control molecule, Ecb, did not cause an increase in C5a binding, suggesting that this was a result of rebinding of C5a to disengaged hC5aR and not, for example, because of an increase in receptor expression. This assay showed that C5a can potentially interact with these cells that are attacked by LukSF. Because the hC5aR expression levels of unstimulated human PMNs were comparatively low (Supplemental Fig. S2) and because these cells express another LukS-binding receptor—hC5aR2—we tested whether the rebinding of anti-CD88 could be detected on these S(wt)-precoated cells in the presence of increasing concentrations of F(G130D) (Supplemental Fig. S9). As a reference also, the rebinding of anti-CD88 on HEK-hC5aR cells was analyzed. As expected, a significant increase on anti-CD88 S(wt)-precoated HEK-hC5aR cells was detected in a 300 nM concentration of F(G130D). Despite low hC5aR expression levels and possible competitive binding of S(wt) by hC5aR2, a slight increase of anti-CD88 was also detected on S(wt)-precoated PMNs. As C5a is a potent anaphylatoxin that is generated during complement activation and potentially plays a crucial role in *S. aureus* infections (55), we next analyzed whether LukSF could lead to complement activation and C5a formation in an *ex vivo* full blood assay. We used soluble C5b-9 as a marker for terminal complement activation and C5a formation, not C5a, as LukS is known to compete with C5a for binding to hC5aR on neutrophils (18). The presence of 200 nM S(wt)F(wt) clearly increased formation of soluble C5b-9 compared with full blood without any toxin or only S(wt) (Fig. 5*D*), indicating that LukSF-mediated cell lysis increases C5a formation and potentially also inflammation in the site of infection.

## DISCUSSION

To determine the stoichiometry of the toxin components without immobilizing the protein on a surface or within a crystal, we used single-molecule imaging of the actual pore formation mechanism within a living cell, including the target receptor crucial for the complex formation. This kind of study on protein complex formation has not been done before, primarily as a result of the difficulty of labeling the components and the high native fluorescence background in mammalian cells. Our covalent labeling strategy and high excitation intensity TIRF microscopy, combined with advanced image analysis tools, open the way for further studies into many other pore-forming toxins and processes involving membrane-bound protein complex formation.

The finding that the toxin complexes are found in receptor clusters indicates that lysis of cells depends on the local density of hC5aRs that will initiate the pore formation process by docking LukS close to the cell membrane, such that 4 hC5aR-LukS dimers (assuming that 1 LukS binds only 1 hC5aR, although hC5aR are randomly clustered on the membrane) can interact with the free, nonbound LukF that will eventually form an octamer (*i.e.*, 4 × 4) and a functional pore with LukS. That hC5aR disassociates from complete pores leaves it free to interact with the C5a formed during complement activation, amplified by LukSF-mediated cell lysis itself. In addition to invading microbes, apoptotic and necrotic cells are known to activate complement (56). The release of locally generated C5a (Fig. 5) and its interaction with adjacent cells, such as endothelial or lung epithelial cells (57), could explain the mechanism behind the exacerbated inflammation characteristics exhibited in necrotizing pneumonia. This is an important finding, suggesting that the cause of infection can dramatically affect the magnitude of the inflammatory response and is highly dependent on the dynamics of microbial molecules interacting with human receptors. In addition, the disengaged hC5aR is possibly available for new toxins to bind; although indirect, our findings suggest that this may allow the receptor to be recycled and reused by additional LukS molecules. Our finding that C5a can rebind provides indirect evidence that additional LukS can also bind.

To characterize the hC5aR interaction with LukSF at a molecular level, we used maleimide-labeled toxins and HEK cells, which expressed only hC5aR and not the second docking target hC5aR2 for LukSF or CD45 for LukF, that are all present on human PMNs (49, 58). We verified that the interaction between maleimide-labeled toxin component mS* and the cell-surface receptor is required for the target recognition and cell lysis similarly, as shown before for S(wt) (18), both for human PMNs and hC5aR-expressing HEK cells that were chosen for TIRF imaging because of their stability and ability to form monolayers on the microscopy coverslip.

With the characterization of the mobility of hC5aR and LukS in live cells, we find that roughly half of hC5aR and LukS foci diffuse relatively freely in the cell membrane, whereas the remainder are confined to zones in the membrane of ∼400 nm effective diameter. However, when LukF is present, >90% of LukS foci become immobile (confined). If LukS were to undergo a conformational change following LukF binding, then this may potentially expose hydrophobic residues that could facilitate insertion of the toxin into the hydrophobic interior foci of the phospholipid bilayer. This stable insertion of the LukSF complex into the cell membrane then leads to pore formation across the whole cell membrane. This hypothesis is strongly supported by the β-barrel prepore-pore formation putative mechanism of γ-hemolysin. Here, the residues responsible for binding with the phospholipid head group are located at the bottom of the rim domain, whereas the stem domain forms an antiparallel β-barrel of which the bottom half comprises the transmembrane portion of the pore (30). This change from the receptor-associated LukS to the cell membrane-associated LukSF complex can be seen as a change in the proportion of mobile (receptor-associated LukS) and immobile (toxin complexes inserted into cell membrane) foci detected in live cells, unlikely to be mobile, as the β-hairpin of LukS and LukF is inserted across the membrane during pore formation and therefore, presumably provides a strong anchor for the formation of a stable membrane-permeating pore. GPCRs similarly are known to have heterogeneous mobility and lateral distribution properties in living cells at different states, for example, before and after activation (59).

Crystallographic evidence from the monomeric LukF and LukS components and the intact γ-hemolysin pore suggests that the pore is octameric, formed from 4-plus-4 LukF/LukS subunits (29, 60, 61). Our findings support this octamer model but unlike previous studies, also indicate that LukS preforms into a tetramer without LukF, and that formation of this tetramer is facilitated by the close proximity C5aR clusters. Based on the earlier scientific evidence, it is known that LukS does not form homodimers, and therefore, it is unlikely that these LukS molecules interact with each other in these clusters (29). Therefore, a more plausible explanation is that LukS are sufficiently, closely packed inside these clusters so as to enable a single LukF to bind to 2 LukS molecules simultaneously on 2 sites of LukF. The presence of LukS tetramers in the absence of LukF cannot be further explained by our data. It is, however, possible that if LukS molecules would be associated as a tetramer when bound on the receptor, then the conformational changes on LukS caused by interactions with LukF should enable association of the LukF subunits to the complex. According to our findings presented here, this scheme is possible, as in these assays, LukS was first enabled to bind to the receptors, and the effect of freshly formed complexes by free, unbound LukS was eliminated by using a washing step before addition of LukF. Each octamer component consists of cap, rim, and stem domains. Here, the cap domain contains the site for LukS/LukF interaction, whereas the stem domain unfolds and forms the transmembrane β-barrel upon pore formation. Within crystallization, the 2-methyl-2,4-pentanediol molecules are bound at the base of the rim domain and recognized by Trp177 and Arg198 residues that may participate in recognition of the phospholipid bilayer, as suggested in a crystal structure of the LukF monomer (62). In contrast, the structure of the γ-hemolysin suggests a membrane-interaction site within residues Tyr117, Phe119, and Phe139 on the same toxin component (29). The crystal structure of Luk components ED, determined recently, reveals important details of the residues on Luk component E required for receptor identification (63). This component corresponds to the receptor-binding component LukS on the LukSF complex, scanning mutagenesis, indicating that LukS residues Arg73, Tyr184, Thr244, His245, and Tyr250 and to a lesser extent, Tyr181, Arg242, and Tyr246 are involved in binding to the neutrophil surface (64).

These results suggest that further binding sites for hC5aR on LukS could be possible in addition to those identified in the LukS rim domain (64). However, as the binding of LukS to neutrophils is inhibited by the C5a, it is likely that LukS has only 1 binding site on the receptor (20). This is also supported by the similar inhibition profiles of LukS and C5a toward anti-CD88 binding on hC5aR shown in this study. Therefore, the association of LukS with ∼4–5 hC5aR molecules could be explained by the previous suggestion that C5aR forms homo-oligomers in living cells (65). Our findings imply that LukSF assembly is dependent on the hC5aR cell-membrane area density as opposed to the effective hC5aR concentration when calculated over the whole of a target cell volume, such that even when hC5aR cellular expression levels are low, for example, when inflammatory mediators are formed to limit the inflammation (50), a cell lysis response may potentially be achieved through the efficient targeting of receptor clusters and putative recycling of the receptor molecules in the cell membrane to be reused by free, nonbound LukS to get engaged in octamer pore formation. It is possible, in principle, that overexpression of hC5aR on HEK cells could lead to an increased ability to form hC5aR clusters, although expression levels are within the range of those in activated neutrophils, exacerbated by the intracellular GFP (although we used the mGFP variant). Other studies, however, have shown that hC5aR forms clusters of homodimers or heterodimers with the second C5aR C5LR (C5aR2) or other GPCRs, such as CCR5, especially under high concentrations of C5a (47, 65, 66).

Previous *in vitro* studies on LukSF pores formed on human leukocytes and rabbit erythrocytes have found evidence for both octamers and hexamers, but importantly, both suggest a LukS:LukF ratio of 1:1 (17, 67, 68). Interestingly, we did not observe any correlation to the number of hC5aR present with LukF incubation time once LukF was already bound to LukS. Moreover, when LukS was incubated with LukF, using sortase-labeled hC5aR cells, a significant reduction was observed in the FRET efficiency signal between LukS and C5aR. It is unlikely that the reduction that was observed in FRET efficiency would be a result of a conformational change, as the cysteine mutation used for maleimide labeling was designed to be exposed on the cap domain of LukS and LukF (Supplemental Fig. S1) that in light of the structural data, undergo minimal or no conformational changes during complex assembly (30). As our biochemical assays indicate that LukF does not bind directly to hC5aR-expressing cells and that binding of LukF to LukS results in an increased distance between the receptor and the complex, this suggests that LukF binding to LukS results in LukS dissociating from the receptor, released as a newly formed LukSF complex.

We cannot directly determine the cause of disassociation in our present study; however, one explanation may lie in the conformational change during the prepore-to-pore transition that has been shown to occur on γ-hemolysin complexes subsequently after binding of LukF to LukS (29, 30). Interestingly, this same study shows that during the prepore state, the space for the transmembrane region is occupied by the rim domain of the adjacent octamer in a LukSF crystal. One explanation for these observations that remains to be explored is that in addition to the stem domain, the residues within the rim domain that interact with the receptor might also have different orientations in the prepore state when compared with the pore state. In addition to using the maleimide-labeled mLukF and mLukS and fluorescence microscopy, the putative dissociation of the hC5aR from the LukSF complex was further verified by using the S(wt) and F(wt) proteins in an assay where LukS-coated hC5aR cells were incubated with increasing concentrations of LukF. Here, an increase in anti-CD88 binding also clearly indicates LukSF dissociation. In all of the assays where we could observe 20–30% receptor dissociation, we used sublytic concentrations of LukF to be able to measure healthy cells with normal membrane fluidity and natural behavior rather than dead cells. This kind of receptor disengagement has been shown before by at least the cytotoxin intermedilysin, which interacts with a GPI-anchored complement regulatory molecule on the cell membrane (69). Moreover, dissociation of the LukSF complex is also supported by electron microscopy of LukSF on human leukocyte membrane fragments. Here, the ring-shaped oligomers with outer 9 nm and inner 3 nm diameters were shown without a receptor (68).

In this study, we also show for the first time to our knowledge that the dissociated receptor can be reused by free, unbound C5a. In our full blood model, we observed that LukSF-mediated cell lysis clearly increased complement activation and C5a formation. The increase in C5a concentration in the site of infection could potentially limit the availability of hC5aR for LukS molecules on neutrophils and thereby, reduce lytic activity of the toxin, as C5a has previously been shown to reduce LukSF-mediated lysis *in vitro* (18). The rebinding of C5a on the receptor may therefore indicate that in natural settings, where all components (*i.e.*, LukS, LukF, and C5a) are present, C5a can outcompete binding of LukSF on the target cells. Therefore, putative recycling of the receptor could be 1 strategy for the toxin to ensure that a sufficient number of pores will damage the cells, especially when a limited number of receptors are available.

**Figure 6:**
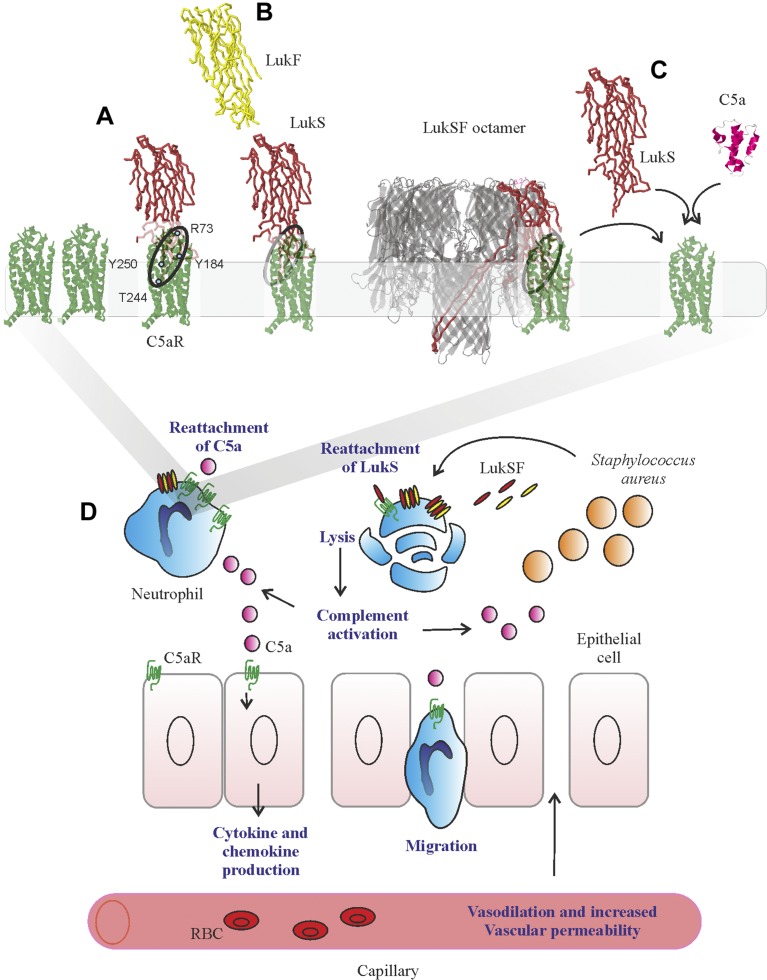
Model for LukSF receptor binding and the mechanism of LukSF-induced inflammation. *A*). LukS [Protein Data Bank identification (PDB ID): 1T5R] binds on hC5aR (structure based on angiotensin receptor data PDB ID: 4YAY as a soluble monomer on the cell membrane. Each LukS monomer binds 1 hC5aR molecule *via* the receptor-interacting residues R73, Y184, Y250, and T244 (marked with blue dots) within a cluster of ∼4–5 hC5aR homo-oligomers Upon binding to hC5aR, LukS exposes residues for LukF (PDB ID: 1LKF) binding (interface indicated by dashed ellipse). In these tight clusters, each LukF can bind to 2 LukS monomers *via* 2 interfaces. *B*) Binding of LukF on LukS and formation of the octameric pore (PDB ID: 3B07) causes dissociation of the receptors from the complex because of leakage of the cell membrane and possibly also because the receptor-binding region (marked with a circle) is buried between the monomers in the complex. *C*) The detached hC5aR molecule can be reused by its ligands LukS or C5a anaphylatoxin (PDB ID: 1KJS). *D*) Zoom-out of *A*–*C*, illustrating the putative mechanism of LukSF-induced inflammation. RBC, red blood cell.

There are several steps on the Luk complex assembly that may be critical for the function of the toxin. Based on our observations, we provide new information on Luk–receptor interactions and propose 2 additional stages to the processes of pore formation and the mechanism by which LukSF potentially induces inflammation (**Fig. 6*A***). Stage 1 is the binding of LukS to hC5aR clusters. The first step in this process is the target recognition of LukS binding to the membrane receptor. Stage 2 is the binding of 4 LukF molecules to 4 LukS molecules, resulting in a hetero-octamer LukSF nanopore in the neutrophil cell membrane. Stage 3 is then the dissociation of the receptors from the LukSF complex, enabling the receptor to be reused for subsequent binding of the free, unbound ligand to generate more nanopores in the cell membrane and enhance the damage to the neutrophil. In addition to previous studies (14), we suggest that LukSF-mediated cell lysis and dissociation from hC5aR can potentially amplify *S. aureus*-mediated inflammation in the site of infection (Fig. 6). The direct lysis of neutrophils is enhanced by newly formed LukSF complexes that are formed on the cell membrane hC5aR *via* reattachment of new LukS. Neutrophil lysis activates the complement system, and the newly generated C5a induces cytokine/chemokine production and neutrophil chemotaxis *via* the C5a/C5aR signaling pathway on adjacent cells. Furthermore, the increased vasodilation and vascular permeability (Fig. 6*B*) leads to massive neutrophil accumulation and tissue injury at the site of bacterial infection (70).

In summary, our findings that the receptors of targeted host cells dissociate rapidly from the Luk complex upon formation of a harmful toxin pore, freeing up mobile receptor seeds that can diffuse to other parts of the cell membrane, suggest a hitherto undiscovered strategy used by microbes to kill human immune cells. This putatively enables a limited number of receptors to be recycled as docking for the Luk or potentially the anaphylatoxin C5a to ensure that enough pores will form to damage the host cell and simultaneously maintain or possibly amplify the inflammation in the site of infection. This discovery may generalize to other bicomponent toxins that use a similar docking receptor, such as the C5aR receptor, including the family of *Staphylococcal* bicomponent Luk of HlgC/HlgB, HlgA/HlgB, LukE/LukD (CXC chemokine receptors 1 and 2 and CCR5), and LukM/LukF′ for bovine CCR2. These results highlight the importance of Luk-receptor interactions in pore formation and may facilitate further understanding of the role of pore-forming toxins in *S. aureus* infections. This new mechanistic insight may prove valuable to the development of future antibacterial and anti-inflammatory therapies, especially important in light of the growing menace of global antimicrobial resistance.

## Supplementary Material

This article includes supplemental data. Please visit *http://www.fasebj.org* to obtain this information.

## Abbreviations

CCR: C–C chemokine receptor
CD88: cluster of differentiation 88
Ecb: extracellular complement binding
F(G130D): G130D leukocidin component F
F(wt): wild-type *Staphylococcus aureus* leukocidin F
FRET: Förster resonance energy transfer
GFP: green fluorescent protein
hC5aR: human C5a receptor
HEK: human embryonic kidney
HSA: human serum albumin
Luk: leukocidin
mF: *Staphylococcus aureus* leukocidin F mutant
mF*: labeled *Staphylococcus aureus* leukocidin F mutant
mGFP: monomeric green fluorescent protein
MRSA: methicillin-resistant *Staphylococcus aureus*
mS: *Staphylococcus aureus* leukocidin S mutant
mS*: labeled *Staphylococcus aureus* leukocidin S mutant
MSD: mean squared displacement
PDB ID: Protein Data Bank identification
PE: phycoerythrin
PFT: pore-forming toxin
PMN: polymorphonuclear cell
pRSET: plasmid Restriction Enzyme T7 promoter
PSF: point spread function
PVL: Panton-Valentine leukocidin
RPMI: Roswell Park Memorial Institute
S(wt): wild-type *Staphylococcus aureus* leukocidin S
SSI: structural similarity index
TIRF: total internal reflection fluorescence
