# Supplementary material for: Staphylococcus aureus toxin LukSF dissociates from its membrane receptor target to enable renewed ligand sequestration
Source: FASEB J. 2018 Dec 3;33(3):3807–24. doi: 10.1096/fj.201801910R (PMC6404581; doi:10.1096/fj.201801910R)
Supplement: Supplementary file 8 [file fj.201801910R.sd1.pdf]

Supplementary Materials

Staphylococcus aureus toxin LukSF dissociates from its membrane receptor target to enable renewed ligand sequestration

Karita Haapasalo[*, †, 2], Adam J. M. Wollman[‡, 2], Carla de Haas[*], Kok van Kessel[*], Jos van Strijp[*], Mark C. Leake[‡, 1, *]

[*] Department of Medical Microbiology, University Medical Center Utrecht, Utrecht University, Utrecht, The Netherlands

[†] Department of Bacteriology and Immunology, and Research Programs Unit, Immunobiology, University of Helsinki, Helsinki, 00014, Finland

[‡] Biological Physical Sciences Institute, Departments of Physics and Biology, University of York, York, YO10 5DD, United Kingdom

[2] These authors contributed equally

[1] Correspondence: Prof Mark Leake, Biological Physical Sciences Institute, Departments of Physics and Biology, University of York, York YO10 5DD, UK. Tel: +44 (0)1904322697. Email: mark.leake@york.ac.uk. Orcid ID**:** http://orcid.org/0000-0002-1715-1249.

[*] Correspondence: Email: mark.leake@york.ac.uk.

Supplemental Figure 1 Construction of recombinant leukocidin proteins. (A) Schematic of TIRF imaging assay. (B) (left panel) Crystal structure of octameric pore complex of γ-hemolysin (PDB ID:3B07). K273/Y111 and K289 on S and F components of γ-hemolysin corresponds to our engineered mutations, K281C/Y113H and K288C, on LukSF marked in their equivalent places on γ-hemolysin; (right panel) SDS-PAGE of the unlabeled LukSK281CY113H and LukFK288C (mS and mF) and Alexa-labeled mS* and mF* toxin components, bands visible at locations consistent with molecular weight of 33 kDa and 34 kDa for LukS and LukF respectively.

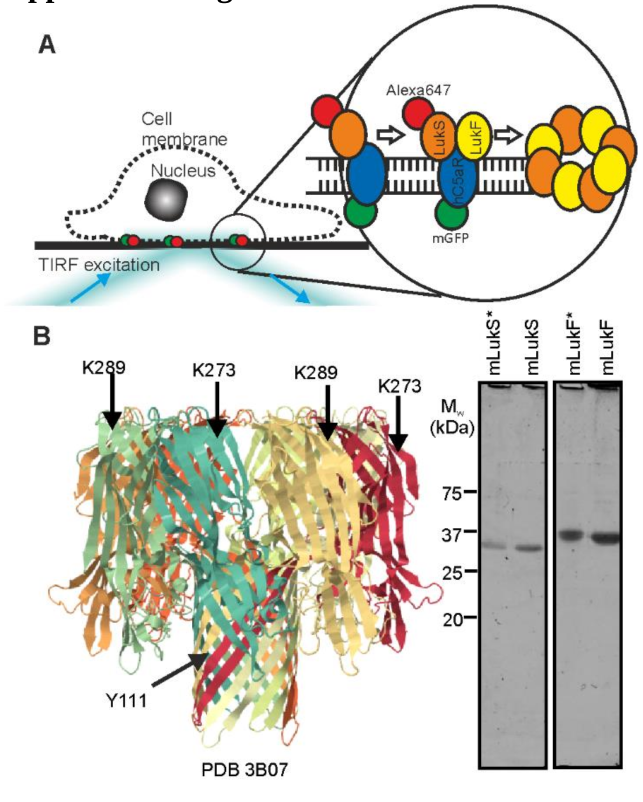

Supplementary Figure 1

Supplemental Figure 2. Expression of hC5aR on cells

To measure hC5aR expression levels on different cell types and conditions PMNs, THP-1 monocytes and PMA activated THP-1 macrophages were analyzed for anti-CD88 binding. The hC5aR expression levels were low compared to the PMA stimulated THP-1 macrophages that showed overlap with the hC5aR expression levels of the used HEK-hC5aR cells. The histogram is a representative of 4 individual experiments.

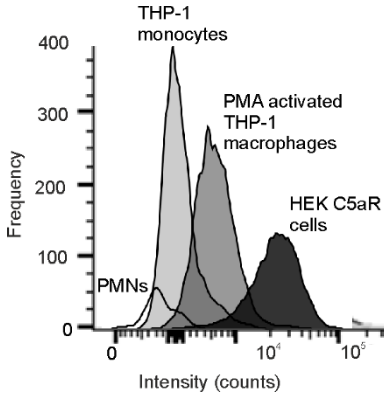

Supplementary Figure 2

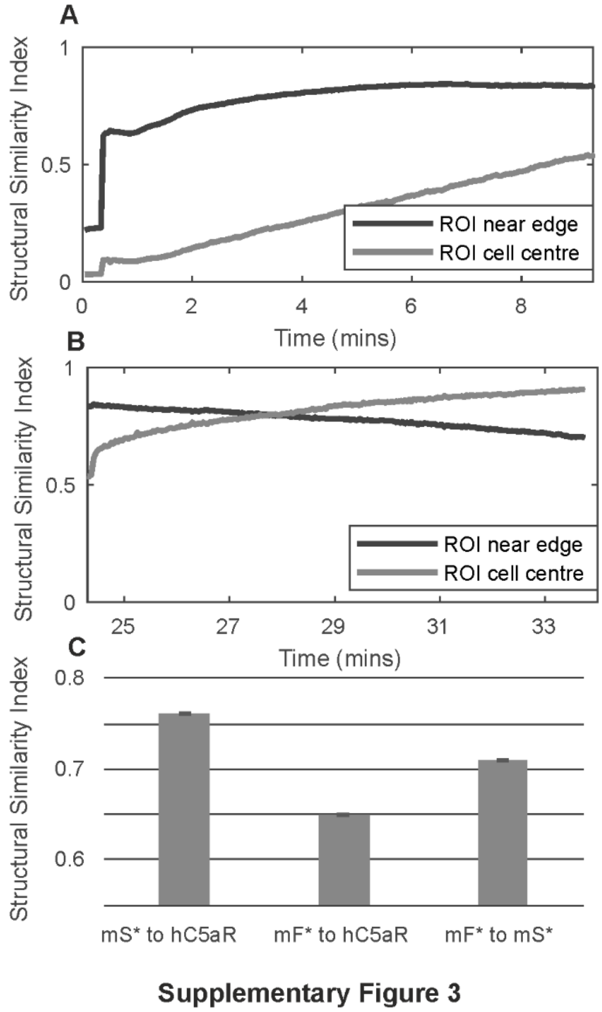

Supplemental Figure 3. Characterization of colocalization of LukS/F with hC5aR form images in Fig. 2 (A) Structural similarity index (SSI) between hC5aR-GFP and mLuks* for the region of interest (ROI) near edge (shown in Fig. 2A) and an ROI in the centre of the cell as a function time during addition of LukS. (B) SSI as (A) after LukF addition. (C) Mean SSI between the 3 channels in Fig. 2C. Standard error in the pixel mean is ~0.1%.

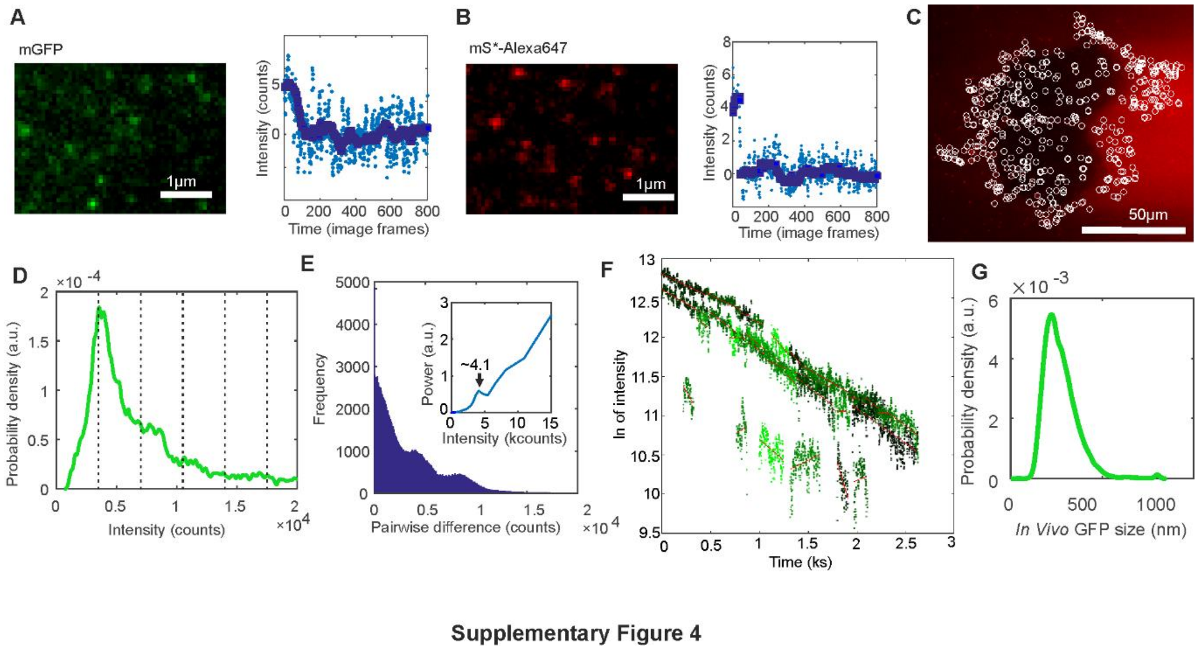

Supplemental Figure 4. Fluorescent protein characterization. A. Fluorescence micrograph of immobilized mGFP and an intensity *vs.* time trace for one foci showing a

single photobleach step. Raw data in light blue and edge-preserving Chung-Kennedy filtered data in dark blue. B. As A for mS*-Alexa647, C. mS*-Alexa647 micrograph (red) with found foci indicated as white circles. D. Intensity distribution of Alexa 647 foci intensities from whole photobleach experiment showing periodicity at ~3,500 counts on our camera detector. (N~1000 foci) E. Pairwise distance distribution of intensity in D with Fourier spectrum (inset) showing peak at ~4,000 counts. F. GFP foci intensity (natural log) time traces (green) with linear fits (red). G. Gaussian sigma fit distribution of GFP foci found towards the end of the photobleach.

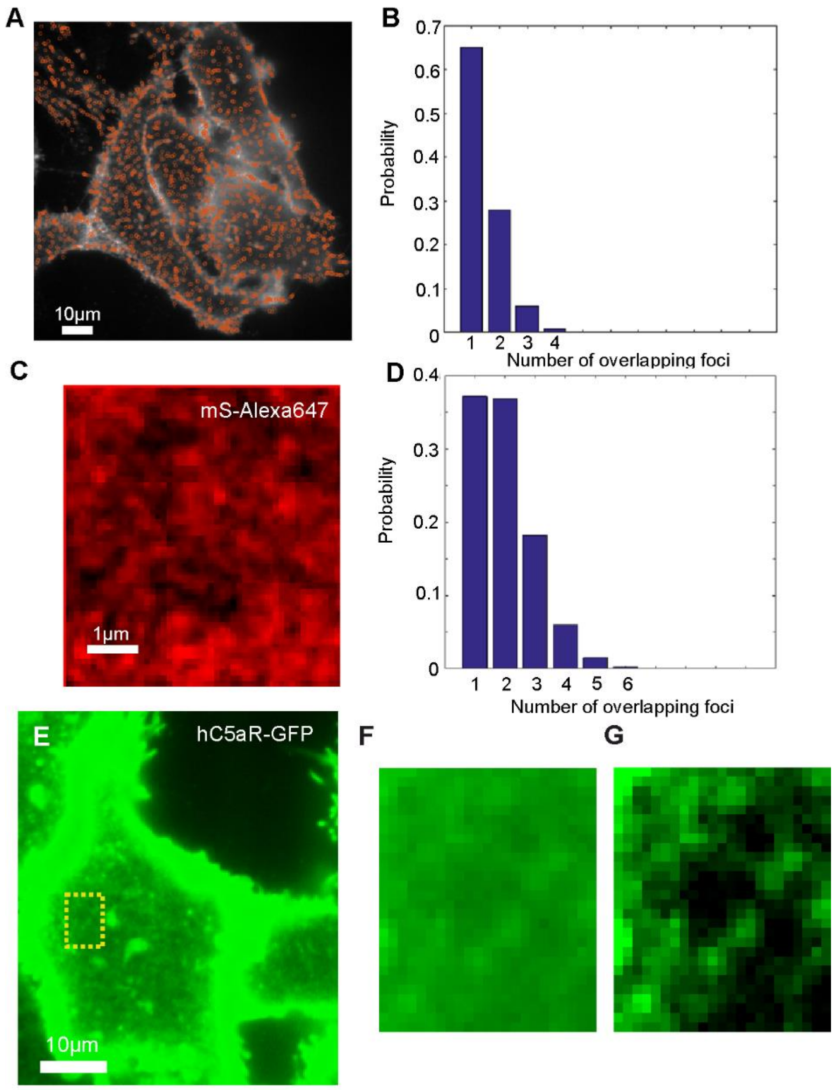

Supplementary Figure 5

Supplemental Figure 5. Density of LukS spots. A. micrograph of mS* (white) with found foci (orange circles) B. Probability distribution of overlap frequency using spots in A to calculate density. C. Zoom in of mS* micrograph. (N~1000 foci) D. Probability distribution of overlap frequency using intensity in C to calculate maximum density estimate. (N~300 foci) E. Fluorescence micrograph of hC5aR-GFP cell. F. zoom in or E. G. same as F. with adjusted levels to highlight clusters.

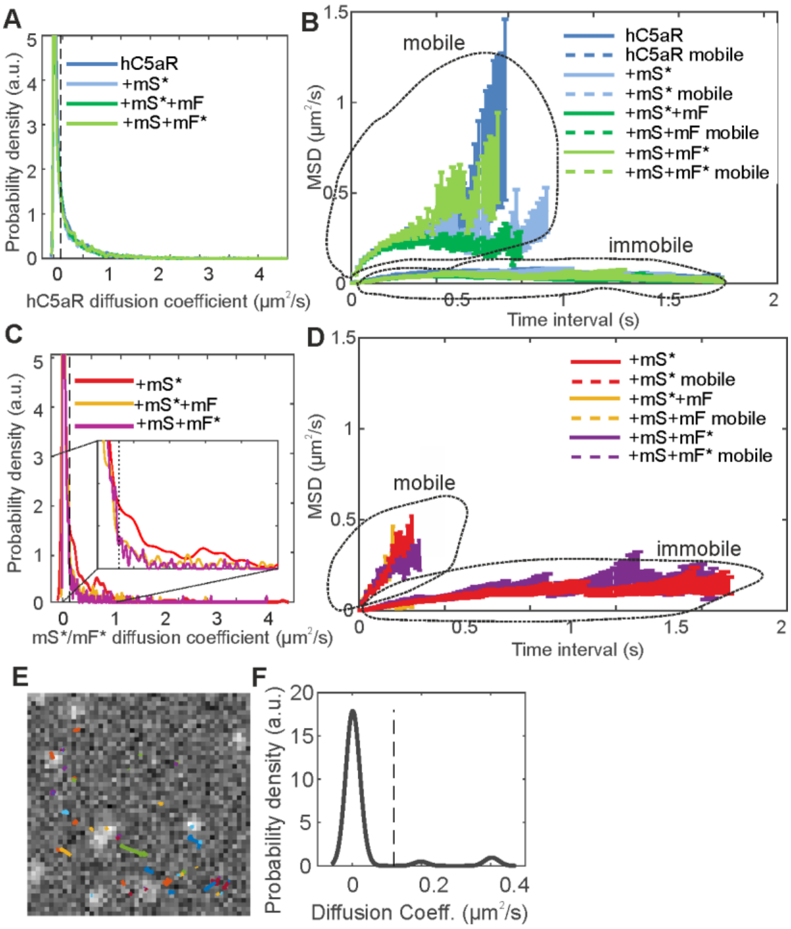

Supplementary Figure 6

Supplemental Figure 6. Mobility analysis. **A.** The probability distribution of microscopic diffusion coefficient showing the threshold for immobility as black dotted line and B. the mean squared displacement against time interval for mobile (upper) and immobile (lower) of hC5aR. C. and D. similar for mS+mF*. An insert showing a zoomed in portion of the plot is shown in C. to better illustrate the division between mobile and immobile. N=20-30 cells, ~1000-10000 foci. E. Simulated image of immobile foci with representative background, noise and foci intensity. Measured trajectories overlaid in colored lines. F. The distribution of diffusion coefficients measured from found trajectories in E, with the *in vivo* immobile threshold shown as a dotted line. N=100 simulated tracks.

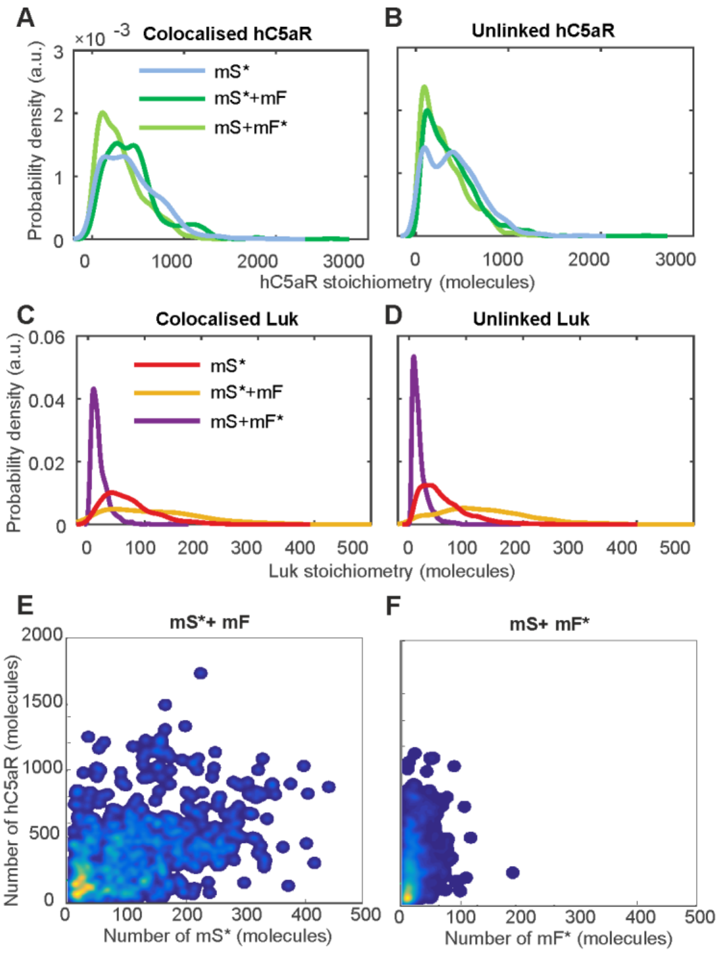

Supplementary Figure 7

Supplemental Figure 7. Colocalization analysis. The probability distribution of linked (A) and unlinked (B) hC5aR and similar for mS* (C. and D.). E. and F. False-color heatmap scatter plots indicating that h5CaR stoichiometry is uncorrelated to mS or mF stoichiometry in the presence of mF. R^2=-0.18 and -0.28 respectively. N~1000 foci from ~10 cells for each.

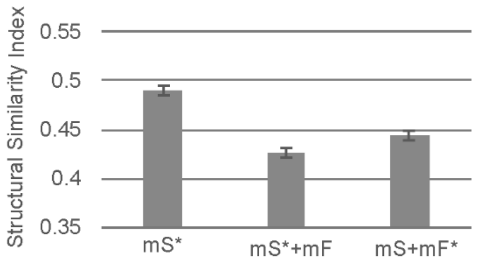

Supplementary Figure 8

Supplemental Figure 8. Fixed cell structural similarity index (A) Micrographs of fixed hC5aR-mGFP HEK cells treated with LukSK281CY113H (mS) and LukFK288C (mF) showing hC5aR-mGFP (left) and Alexa647 (middle) and merge (right) on Alexa-labeled mF (mF*) (B) The mean SSI between mS* or mF* and hC5aR. Standard error in the mean from 7 cells.

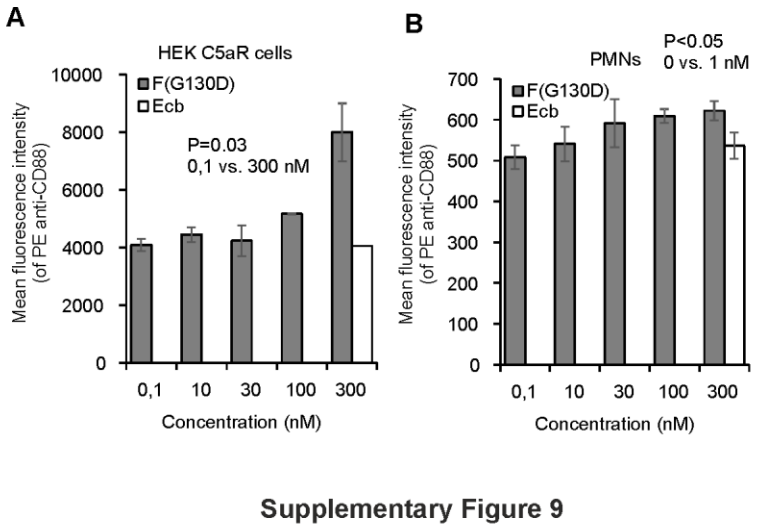

Supplemental Figure 9. LukSF dissociation on hC5aR expressing HEK cells and PMNs. (B) Disengagement of hC5aR from LukSF was observed on both HEK-hC5aR cells (A) and PMNs (B) as an increase in PE conjugated anti-CD88 binding (right vertical axis, indicated with bars) on S(wt) pre-coated cells using increasing concentrations (horizontal axis) of non lysing LukF mutant (FG130D). Ecb was used as negative control in the assays. Statistical significances are calculated using Student's *t*-test (n=2). Error bars indicate SD.

Supplemental Movie 1. Deposition of mS on hC5aR-mGFP HEK cells. The movie is shown in two clips before (no toxin) and after addition of Alexa 595 labeled mS (add mS*). This movie was recorded for *ca.* 13 min and displayed here at 100x speed.

Supplemental Movie 2. (.avi). Lysis of hC5aR-mGFP HEK cells incubated with Alexa594 labeled mS* and mF. The cells were preincubated with mS* and the lysis of the cells were monitored for *ca.* 13 min after addition of mF (add mLukF). The red arrow points to the vesicles released during cell lysis. This movie was recorded for *ca.* 13 min and displayed here at 100x speed.

Supplemental Movie 3. (.avi). Colocalization of Alexa594- and Alexa647-labeled mF and mS (mF*Alexa594 (blue) and mS*Alexa647 (red)) with hC5aR-mGFP (green) on HEK cells. This movie was recorded for *ca.* 13 min and displayed here at 40x speed.

Supplemental Movie 4 (.avi). Imaging live hC5aR-mGFP cells. After 1-2 min of exposure, several distinct, mobile, circular fluorescent foci in the planer membrane regions were observed. Movie is displayed in real time.

Supplemental Movie 5 (.avi). Imaging mS* incubated with hC5aR-mGFP cells. Several distinct, mobile, circular fluorescent foci were observed. Movie is displayed in real time.

Supplemental Movie 6 (.avi). Imaging mS* incubated with hC5aR-mGFP cells, zoomed in and sped up to highlight differences in mobility. Scale bar 2µm.

Supplemental Movie 7 (.avi). Imaging mF* incubated with mS and hC5aR-mGFP cells, zoomed in and sped up to highlight differences in mobility. Scale bar 2µm.
